# Neuronal Expression of Glucosylceramide Synthase in Central Nervous System Regulates Body Weight and Energy Homeostasis

**DOI:** 10.1371/journal.pbio.1001506

**Published:** 2013-03-12

**Authors:** Viola Nordström, Monja Willershäuser, Silke Herzer, Jan Rozman, Oliver von Bohlen und Halbach, Sascha Meldner, Ulrike Rothermel, Sylvia Kaden, Fabian C. Roth, Clemens Waldeck, Norbert Gretz, Martin Hrabě de Angelis, Andreas Draguhn, Martin Klingenspor, Hermann-Josef Gröne, Richard Jennemann

**Affiliations:** 1 Department of Cellular and Molecular Pathology, German Cancer Research Center, Heidelberg, Germany; 2 German Mouse Clinic, Institute of Experimental Genetics, Helmholtz Zentrum München, Neuherberg, Germany; 3 Molecular Nutritional Medicine, Else-Kröner Fresenius Center, Technische Universität München, Freising-Weihenstephan, Germany; 4 Institute for Anatomy and Cell Biology, University of Greifswald, Greifswald, Germany; 5 Institute for Physiology and Pathophysiology, Heidelberg University, Heidelberg, Germany; 6 Medical Research Center, Heidelberg University, Heidelberg, Germany; University of Cambridge, United Kingdom

## Abstract

Hypothalamic neurons are main regulators of energy homeostasis. Neuronal function essentially depends on plasma membrane-located gangliosides. The present work demonstrates that hypothalamic integration of metabolic signals requires neuronal expression of glucosylceramide synthase (GCS; UDP-glucose:ceramide glucosyltransferase). As a major mechanism of central nervous system (CNS) metabolic control, we demonstrate that GCS-derived gangliosides interacting with leptin receptors (ObR) in the neuronal membrane modulate leptin-stimulated formation of signaling metabolites in hypothalamic neurons. Furthermore, ganglioside-depleted hypothalamic neurons fail to adapt their activity (c-Fos) in response to alterations in peripheral energy signals. Consequently, mice with inducible forebrain neuron-specific deletion of the UDP-glucose:ceramide glucosyltransferase gene (*Ugcg*) display obesity, hypothermia, and lower sympathetic activity. Recombinant adeno-associated virus (rAAV)-mediated *Ugcg* delivery to the arcuate nucleus (Arc) significantly ameliorated obesity, specifying gangliosides as seminal components for hypothalamic regulation of body energy homeostasis.

## Abstract 2

Body weight and energy homeostasis are regulated by leptin receptor interactions with gangliosides, a class of plasma membrane lipids, in forebrain neurons of mice.

## Author Summary

Obesity is a growing health threat that affects nearly half a billion people worldwide, and its incidence rates in lower income countries are rising dramatically. As obesity is a major risk factor for type II diabetes and cardiovascular disease, significant effort has been put into the exploration of causes, prevention, and potential treatment. Recent research has demonstrated that a region of the brain called the hypothalamus is a major integrator of metabolic and nutrient signals, adapting food intake and energy expenditure to current metabolic needs. Leptin or insulin receptors located in the plasma cell membrane of neurons sense energy signals from the body. They transmit this information inside the cell, which then regulates neuronal function. In this study, we show that leptin receptors interact with gangliosides, a class of plasma membrane lipids. This interaction is a prerequisite for proper receptor activation. Consequently, ganglioside loss in hypothalamic neurons inhibits leptin receptor signal transduction in response to energy metabolites. Furthermore, mice lacking gangliosides in distinct forebrain areas, amongst them the hypothalamus, develop progressive obesity and hypothermia. Our results suggest a previously unknown regulatory mechanism of plasma membrane lipids for hypothalamic control of body weight.

## Introduction

The investigation of pathogenetic mechanisms underlying obesity has attained significant interest, as obesity has become an endemic metabolic disturbance worldwide. Elevated peripheral energy storage can develop as a consequence of alterations in the neuronal feedback circuits regulating energy homeostasis. The hypothalamus is the main CNS integrator of peripheral energy signals, matching energy intake to energy expenditure for body weight maintenance [1].

Among the most extensively studied peripheral molecules involved in regulating energy homeostasis and feeding behavior in the CNS are the adipocyte-derived hormone leptin as well as insulin [2],[3]. Among numerous leptin- and insulin-sensitive brain areas, the hypothalamic Arc is one of the main regions integrating peripheral energy signals and initiating adaptive metabolic and behavioral responses [4].

Recently, several CNS regions targeted by leptin have emerged that are involved in the regulation of energy metabolism, such as the brain stem nucleus of the solitary tract (NTS) and reward circuits involving the ventral tegmental area [5],[6]. Still, leptin is suggested to exert anti-obesity effects by signaling through “long form” leptin receptors (ObR) abundantly present on both orexigenic neuropeptide Y (NPY)/agouti-related peptide (AgRP) neurons and anorexigenic pro-opiomelanocortin (POMC) neurons in the Arc. Excess NPY signaling abates sympathetically mediated thermogenesis, thereby reducing energy expenditure [7]. NPY and AgRP expression is attenuated upon ObR-induced phosphatidylinositol-3-OH-kinase (PI3k) signaling [8]. Conversely, leptin stimulates the expression of the POMC-derived neurotransmitter α-melanocyte-stimulating hormone (α-MSH) through the Janus kinase/signal transducer and activator of transcription (Jak-Stat) pathway [9]. Alpha-MSH, a potent agonist of melanocortin receptors, inhibits food intake and stimulates the expenditure of excess energy in the body, thus preventing obesity development [10].

Insulin exerts its anorexigenic effects in hypothalamic neurons by directly stimulating insulin receptor autophosphorylation and activation of PI3k. Even though both insulin and leptin receptor stimulation leads to activation of PI3k and subsequent formation of phosphatidylinositol (3,4,5)-triphosphate (PIP3) [11], it has been shown that both hormones exert converging direct actions on POMC neurons, while having opposite effects on AgRP/NPY neurons [12].

GCS is the key enzyme for the biosynthesis of glycosphingolipids (GSLs) and gangliosides, a class of acidic GSLs abundantly expressed by neurons and glial cells [13],[14]. Ganglioside-depleted neurons are viable and show apoptosis rates comparable to wild-type neurons [15]. GSLs including gangliosides contribute to the formation of membrane microdomains, which are important mediators of intracellular signal transduction [16]. GCS expression is crucial for initial postnatal brain maturation and *Ugcg* ^f/f//NesCre^ mice with constitutive *Ugcg* deletion in brain tissue under the control of the nestin promoter die within 3 wk after birth [15]. In 2003, it was shown that GM3 synthase-deficient mice are more sensitive to insulin, thereby protecting these mice from high-fat-diet-induced insulin resistance [17]. A different ganglioside species, GD1a, has been shown to exert activating effects on tyrosine kinase receptors [18]. To address the functional role of GCS in neuronal regulation of energy homeostasis, we have generated and characterized mice with inducible neuron-specific *Ugcg* deletion in adult mouse CNS (*Ugcg* ^f/f//CamKCreERT2^ mice). Cre activity in this mouse model was restricted to distinct populations of forebrain neurons. Hypothalamic nuclei involved in the regulation of energy homeostasis were targeted by this approach. Explicitly, Cre activity was absent in the brain stem NTS, which also contributes to regulation of energy homeostasis.

The present study highlights GCS-derived gangliosides as mediators for ObR-dependent signal transduction at the hypothalamic neuronal membrane. GCS-depleted neurons failed to show ObR activation upon leptin stimulation. Major neuronal gangliosides GM1 and GD1a were recruited to ObR upon ligand stimulation and subsequent signal transduction depended on ganglioside expression in hypothalamic neurons. *Ugcg* ^f/f//CamKCreERT2^ mice deficient in GSLs in hypothalamus developed progressive obesity and decreased sympathetically mediated thermogenesis. rAAV-mediated *Ugcg* delivery to the hypothalamic Arc with ensuing nucleus-specific GSL synthesis significantly ameliorated obesity.

## Results

### Ganglioside Depletion in Cre-Targeted Neurons in Vivo and in Vitro

**Figure 1:**
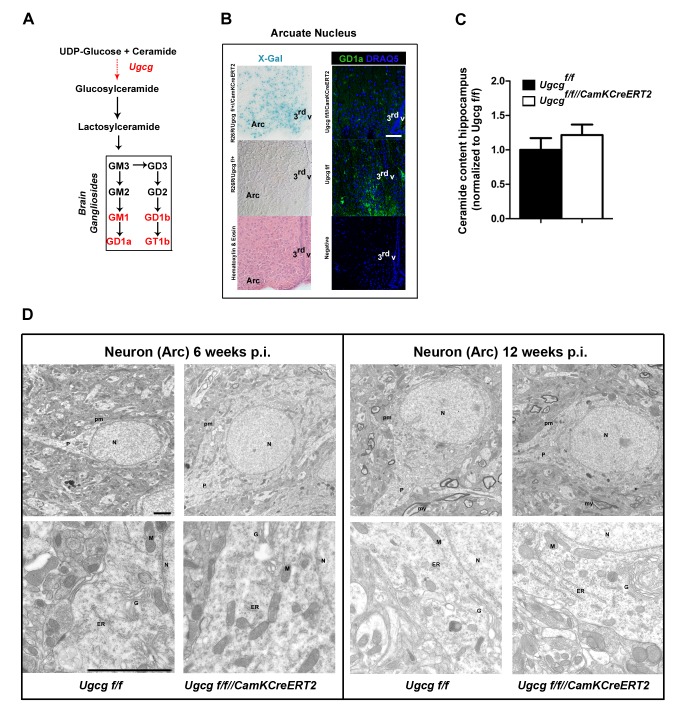
Normal ultrastructure in ganglioside-depleted neurons. (A) Major pathway for biosynthesis of GSL including gangliosides in the brain. (B) X-Gal staining in brains of *R26R/Ugcg* ^f/+//CamKCreERT2^ reporter mice revealed strong Cre activity in the hypothalamic Arc. GD1a immunofluorescence visualized ganglioside depletion in the Arc of *Ugcg* ^f/f//CamKCreERT2^ mice 6 wk p.i. Scale bar: 75 µm. (C) Ceramide levels were not significantly altered in hippocampus of *Ugcg* ^f/f//CamKCreERT2^ mice. Quantification from densitometry analysis of TLC results is depicted (*n* = 3). (D) Neurons in the Arc of *Ugcg* ^f/f//CamKCreERT2^ mice showed normal ultrastructural morphology of plasma membrane (pm), nucleus (N), mitochondria (M), endoplasmic reticulum (ER), golgi (G), projections (P), and myelin sheaths (my) 6 and 12 wk p.i. Scale bar: 2 µm. 3^rd^v, third ventricle.

*Ugcg* ^flox/flox^ (*Ugcg* ^f/f^) mice were bred with mice expressing the inducible CreERT2 recombinase under the control of the Calcium/Calmodulin-dependent Kinase II-*alpha* (CamK) promoter, resulting in forebrain neuron-specific *Ugcg* deletion (*Ugcg* ^f/f//CamKCreERT2^) followed by ganglioside depletion after tamoxifen injection (Figure 1A). Generation of *Ugcg* ^f/f^ mice and CamKCreERT2 mice has been described earlier [15],[19].

Beta-galactosidase (X-Gal) staining of brains from *R26R/Ugcg* ^f/+//CamKCreERT2^ reporter mice indicated strong Cre activity in distinct hypothalamic nuclei, namely in the Arc (Figures 1B and S1B), in the paraventricular nucleus, and in median preoptic area (MnPO) (Figure S1A,B). Additional Cre activity was detected in the lateral hypothalamic area (LHA), in hippocampus, and in the cerebral cortex (Figure S1A,B). Notably, Cre activity was absent in the ventromedial hypothalamus and the NTS in the brain stem (Figure S1A,B). Ganglioside depletion was confirmed in Cre-targeted areas by GD1a immunofluorescence, whereas non-targeted areas retained GD1a expression (Figure 1B and Figure S1A).

Consistent with the expected Cre-activity pattern, in situ hybridization showed *Ugcg* mRNA depletion in hippocampus, cerebral cortex, amygdala, as well as hypothalamic nuclei (Figure S1C). Recombination events were absent in peripheral organs and peripheral nervous tissue (Figure S1D).

Neuron-dense total hippocampi showed significant and stable ganglioside reduction 3 wk postinduction (p.i.), as assessed by thin layer chromatography (TLC) (Figure S1E). Residual gangliosides in the dissected tissue were assumed to result from glial cells as well as from innervating nerve fibers emerging from nontargeted neurons [14]. Ceramide levels in Cre-targeted neuronal populations were unchanged (Figure 1C), and a slight increase in sphingomyelin could be detected (Figure S1F).

In order to investigate if ganglioside depletion abated general neuronal function and integrity in *Ugcg* ^f/f//CamKCreERT2^ mice, both electron microscopy and electrophysiological slice recordings were done at late time points p.i. Electron microscopy from Arc neurons displayed normal ultrastructure of the neuronal nucleus, organelles, and an intact, regular plasma membrane of *Ugcg* ^f/f//CamKCreERT2^ mice both 6 and 12 wk p.i. (Figure 1D). Basic biophysical parameters [spontaneous firing rate, action potential (AP) width, and AP rate of rise] from slice recordings of Arc neurons 12 wk p.i. were unaltered (Figure S2A). The resting membrane potential and the AP threshold were marginally increased in *Ugcg* ^f/f//CamKCreERT2^ mice, however not to an extent that impairs neuronal function (Figure S2B).

In order to confirm these findings in vitro, immortalized mouse hypothalamic cells (N-41 cells) expressing GCS-derived gangliosides (Figure S3A,B) were treated with n-butyldeoxynojirimycin (NB-DNJ) specifically inhibiting GCS [20]. NB-DNJ treatment resulted in approximately 80%–90% ganglioside depletion (Figure S3C). Consistent with the findings in *Ugcg* ^f/f//CamKCreERT2^ mice, membrane integrity and normal cellular ultrastructure of ganglioside-depleted N-41 cells was confirmed by electron microscopy (Figure S3D). Additionally, passive and active membrane properties of cultured primary GCS-deficient hypothalamic *Ugcg* ^f/f//NesCre^ neurons [15] were examined by whole-cell recordings. There were no differences toward control cells in membrane resistance, capacitance, and resting potential (Figure S3E). Spikes evoked by somatic current injection had unaltered threshold, amplitude, and duration (Figure S3F). These results indicate that basic neuronal integrity and general function are not affected by *Ugcg* deletion and subsequent lack of plasma membrane gangliosides.

### Progressive Body Weight Gain, Hypometabolism, and Hypothermia in *Ugcg* ^f/f//CamKCreERT2^ Mice

**Figure 2:**
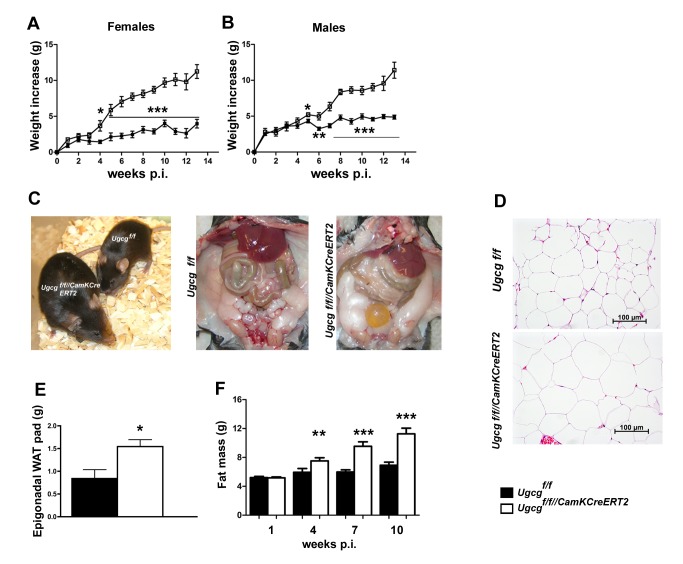
*Ugcg* ^f/f//CamKCreERT2^ mice develop progressive obesity. Both female (A) and male (B) *Ugcg* ^f/f//CamKCreERT2^ mice showed a progressive increase in body weight after tamoxifen induction (*n* = 6–9). (C) *Ugcg* ^f/f//CamKCreERT2^ mice were larger than *Ugcg* ^f/f^ littermates (16 wk p.i.), and body fat mass was prominently elevated. (D) Enlarged adipocytes in *Ugcg* ^f/f//CamKCreERT2^ mice 9 wk p.i. (E) Increased weight of epigonadal WAT 9 wk p.i. in *Ugcg* ^f/f//CamKCreERT2^ mice (*n* = 4–5). (F) NMR analysis revealed significant and progressive accumulation of body fat mass in *Ugcg* ^f/f//CamKCreERT2^ mice (*n* = 9–10). *p≤0.05; **p≤0.01;***p≤0.001. Means ± SEM.

Coinciding with neuronal ganglioside depletion 3 wk p.i. female and male *Ugcg* ^f/f//CamKCreERT2^ mice displayed progressive body weight increase (Figure 2A,B). This phenotype was not detected in heterozygous mice (Figure S4A), as residual GCS activity accounted for maintenance of neuronal ganglioside biosynthesis [15]. *Ugcg* ^f/f//CamKCreERT2^ mice were larger than control littermates 16 wk p.i. (Figure 2C).

Hematoxylin and eosin (HE) staining revealed enlarged adipocytes in epigonadal white adipose tissue (WAT) (Figure 2D). In line with this, epigonadal WAT pad weight was significantly elevated (Figure 2E). Whole body nuclear magnetic resonance (NMR) analysis revealed that body weight increase was due to progressive accumulation of body fat (Figure 2F); lean mass was only marginally elevated 4 wk p.i. (Figure S4B). Adjusted for body weight the initial increase of fat and lean mass was proportional, whereas at later stages fat mass overrode lean mass gain (Figure S4C). Liver steatosis and morphological changes in major peripheral organs of obese *Ugcg* ^f/f//CamKCreERT2^ mice were not detected 9 wk p.i. (Figure S4D). Serum enzyme activities indicative for liver function (glutamate dehydrogenase, glutamic oxaloacetic transaminase, and glutamic pyruvic transaminase) were unaltered (Figure S4E). Likewise, serum cholesterol, urea, glucose, and creatinine did not show any biologically relevant abnormalities (Figure S4F). Coincident with obesity, *Ugcg* ^f/f//CamKCreERT2^ mice were less glucose tolerant than *Ugcg* ^f/f^ mice 12 wk p.i. (Figure S4G) and insulin sensitivity was marginally impaired 10 wk p.i. (Figure S4H). These results demonstrate that *Ugcg* ^f/f//CamKCreERT2^ mice develop progressive obesity that is evident in all adipose compartments with constant lean mass and a shift in body composition toward fat accumulation.

**Figure 3:**
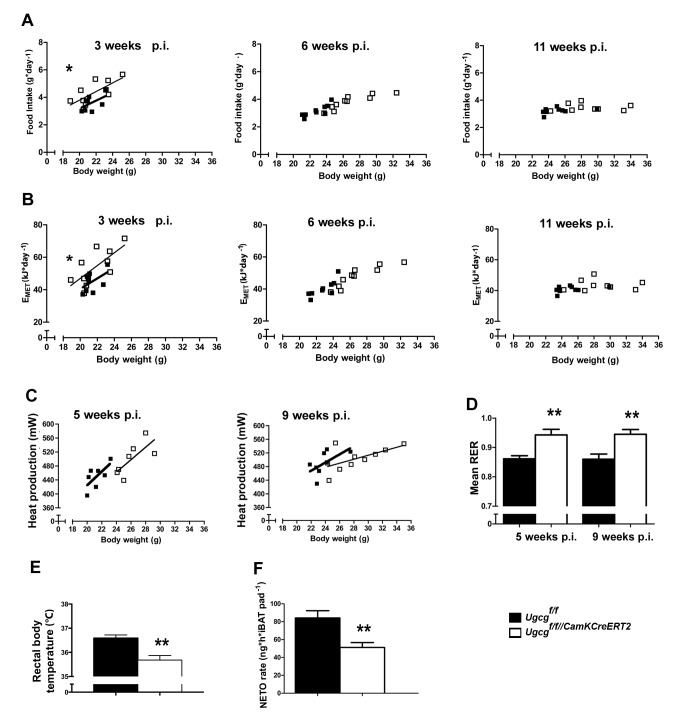
Obese *Ugcg* ^f/f//CamKCreERT2^ mice are initially slightly hyperphagic and show hypometabolism and hypothermia. (A) Food intake per day plotted against body weight of *Ugcg* ^f/f//CamKCreERT2^ mice was slightly but significantly increased 3 wk p.i. and returned to levels not significantly different from control littermates 6 and 11 wk p.i. (*n* = 9) (LM). Individual means. (B) Metabolizable energy per day plotted against body weight in *Ugcg* ^f/f//CamKCreERT2^ mice was slightly increased 3 wk p.i. and returned to normal levels 6 and 11 wk p.i. (*n* = 9) (LM). Individual means. (C) Metabolic rate of *Ugcg* ^f/f//CamKCreERT2^ mice was decreased 5 and 9 wk p.i., indicating lower energy expenditure (*n* = 7–10); *p* = 0.073 week 5; *p* = 0.77 week 11 (LM). Individual means. (D) Mean respiratory exchange rate (RER), as determined during a 21-h indirect calorimetry measurement, was significantly elevated in *Ugcg* ^f/f//CamKCreERT2^ mice 5 and 9 wk p.i., indicating reduced lipid oxidation (*n* = 7–10). (E) Rectal temperature measurements showed a drop in body temperature in *Ugcg* ^f/f//CamKCreERT2^ mice (10 wk p.i.; *n* = 9–10). (F) *Ugcg* ^f/f//CamKCreERT2^ mice showed lower sympathetic activity, indicated by lower norepinephrine turnover (NETO) rate, in total iBAT pad 9 wk p.i. (*n* = 4). *p≤0.05; **p≤0.01;***p≤0.001. Means ± SEM unless stated otherwise.

As tight regulation of energy homeostasis is crucial for body weight maintenance [1], a metabolic characterization was carried out in order to study the relation of energy intake to energy expenditure. Food intake and metabolizable energy (E_MET_) adjusted to body weight were slightly elevated in *Ugcg* ^f/f//CamKCreERT2^ mice before the onset of obesity 3 wk p.i. (Figure 3A,B) when gangliosides were already depleted in Cre-targeted brain regions. Hyperphagia was no longer evident 6 and 11 wk p.i., as food intake and E_MET_ were simply elevated due to higher body weight (Figure 3A,B). Fecal excretion of free fatty acids (FFAs) as well as energy content of feces and extraction efficiency from the food (Figure S5A) were unaltered. Thus, abnormalities in food intake do initially contribute to obesity development, but not for obesity maintenance.

Energy expenditure was monitored by indirect calorimetry for 21 h. Before onset of body weight gain, the metabolic rate was indistinguishable from *Ugcg* ^f/f^ mice 2 wk p.i. (Figure S5B). When adjusted for body weight, the average metabolic rate tended to be lower in *Ugcg* ^f/f//CamKCreERT2^ mice at 5 and 9 wk p.i. (Figure 3C). Spontaneous locomotor activity is one contributor to daily energy expenditure and has been reported to be decreased in obese rodents [21]. However, both before the onset of weight gain and during progressive adiposity, spontaneous open field activity of *Ugcg* ^f/f//CamKCreERT2^ mice was indistinguishable from control littermates (Figure S5C).

The respiratory exchange ratio (RER) provides information on metabolic fuel preferences [22]. *Ugcg* ^f/f//CamKCreERT2^ mice displayed significantly elevated average daily RER values (Figure 3D). This finding suggests a shift from lipid oxidation toward lipid storage [22]. In line with this, fat mobilization in response to fasting as assessed by measuring plasma nonesterified free fatty acids (NEFAs) was impaired. Significantly decreased plasma NEFAs were detected in *Ugcg* ^f/f//CamKCreERT2^ mice 11 wk p.i. (Figure S5D), suggesting a reduced capability to mobilize lipid stores when challenged by food withdrawal.

After the onset of weight gain, *Ugcg* ^f/f//CamKCreERT2^ mice displayed a prominent drop in core body temperature, as exemplarily depicted 10 wk p.i. (Figure 3E). Adipocytes in intrascapular brown adipose tissue (iBAT) were enlarged (Figure S6A), suggesting reduced triglyceride turnover. Ultrastructural analysis of iBAT furthermore revealed mitochondrial disorganization as well as a lower average mitochondrial size (Figure S6B,C). Thermogenesis in iBAT is regulated by synergistic actions of thyroid hormones and sympathoadrenergic signaling [23]. Free triiodothyronine (fT3) and free thyroxine (fT4) levels were normal in *Ugcg* ^f/f//CamKCreERT2^ mice (Figure S6D,E). Thus, thyroid dysfunction was unlikely to account for inappropriate thermoregulation. Decreased sympathetic outflow to adipose tissue is assumed to be associated with impaired lipid mobilization [24]. In fact, both iBAT sympathetic activity, as assessed by norepinephrine (NE) turnover rate (Figure 3F, Figure S6F), and NE content (Figure S6G) were decreased in *Ugcg* ^f/f//CamKCreERT2^ mice.

These results demonstrate that *Ugcg* ^f/f//CamKCreERT2^ mice develop progressive obesity and a shift in body composition toward fat accumulation initially supported by hyperphagia, but maintained due to hypometabolism and hypothermia.

### Reconstitution of *Ugcg* Gene Expression in the Hypothalamic Arc Ameliorates Obesity

**Figure 4:**
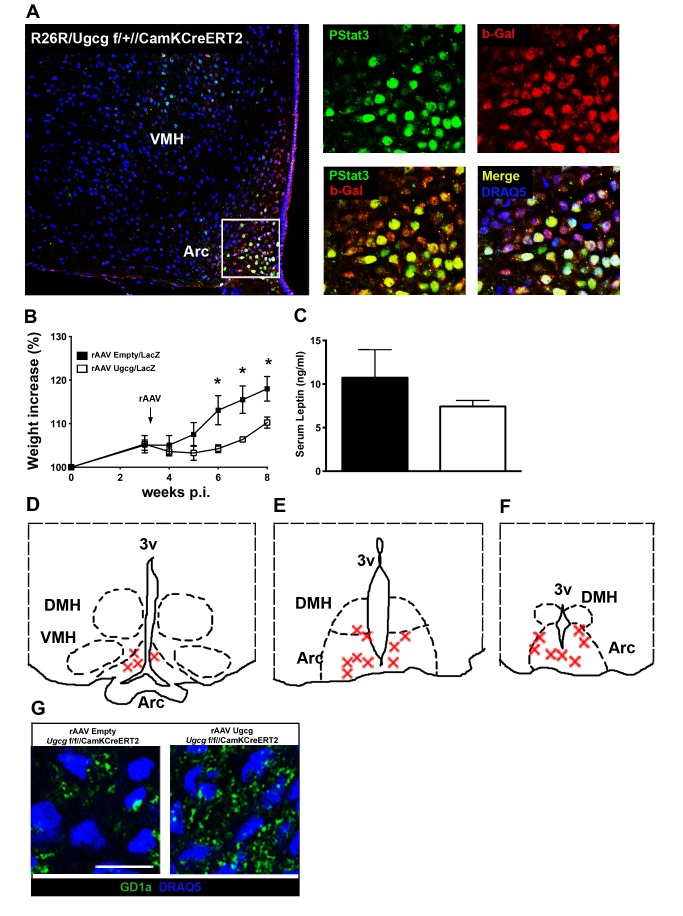
rAAV-mediated *Ugcg* gene delivery to the hypothalamic Arc ameliorates obesity and hyperleptinemia in *Ugcg* ^f/f//CamKCreERT2^ mice. (A) Double immunofluorescence showed that Cre activity, indicated by beta galactosidase staining (b-gal), was targeted to Arc neurons expressing the long form of the ObR, as indicated by PStat3 staining in leptin-injected *R26R/Ugcg* ^f/+//CamKCreERT2^ mice (5 mg/kg leptin, 120 min). (B) Stereotactic delivery of rAA viruses encoding *Ugcg* and *lacZ* to the Arc of *Ugcg* ^f/f//CamKCreERT2^ mice resulted in a significant amelioration in body weight increase compared to rAAV-Empty/lacZ-injected *Ugcg* ^f/f//CamKCreERT2^ mice (*n* = 6–8). (C) Serum leptin tended to be lower in rAAV-Ugcg/lacZ-injected *Ugcg* ^f/f//CamKCreERT2^ mice 8 wk p.i. (*n* = 6–8). (D–F) Targeting of rAAV Ugcg/lacZ- and rAAV Empty/lacZ-injected animals that were included in the analyses. At the end of the experiments, brains were removed and stained for X-Gal to indicate vector delivery. Red marks depict exemplarily areas of strong X-Gal staining in animals considered as Arc targeted. Depicted are areas between bregma −1.9 (D), bregma −2.1 (E), and bregma −2.3 (F). (G) Restored ganglioside biosynthesis in the Arc of rAAV-Ugcg-injected *Ugcg* ^f/f//CamKCreERT2^ mice, as shown by GD1a immunofluorescence 8 wk p.i. Scale bar: 18 µm. *p≤0.05. Means ± SEM.

Several distinct hypothalamic and nonhypothalamic brain regions were targeted by Cre activity in *Ugcg* ^f/f//CamKCreERT2^ mice. Arc neurons in *Ugcg* ^f/f//CamKCreERT2^ mice expressing the long form of the ObR were targeted by Cre activity, as demonstrated by co-immunofluorescence of PStat3 and beta-galactosidase (b-Gal) in *R26R/Ugcg* ^f/+//CamKCreERT2^ reporter mice (Figure 4A). Other leptin-responsive neurons outside the Arc also targeted by Cre activity, such as the MnPO are likely in part contributing to the observed phenotype. However, ObR-expressing neurons in the LHA seem to be recessed by Cre activity (Figure S7A,B).

In order to furthermore clarify the role of the Arc in obesity development, we injected recombinant adeno-associated viruses encoding either *Ugcg* and *lacZ* (rAAV-Ugcg/LacZ) or only *lacZ* (rAAV-Empty/LacZ) bilaterally into the Arc of *Ugcg* ^f/f//CamKCreERT2^ mice after ganglioside depletion before 4 wk p.i. Injection of rAAV-Ugcg/LacZ significantly ameliorated obesity, underlining the importance of *Ugcg* expression in the Arc for body weight maintenance (Figure 4B). Consistently, serum leptin levels tended to be lower in rAAV-Ugcg/LacZ-treated mice (Figure 4C). We verified correct targeting of the Arc by X-Gal staining of the brains injected with rAAV-Ugcg/LacZ and displayed targeted regions in a schematic drawing as well as a typical staining (Figures 4D–F and S7C). Animals that were not targeted by rAAV-Ugcg/LacZ in the Arc (rAAV-Ugcg/LacZ missed) did not improve their weight gain (Figure S7D). Restored ganglioside biosynthesis in the Arc of rAAV-Ugcg-treated animals compared to mice injected with viruses encoding empty plasmid was demonstrated by GD1a immunofluorescence (Figure 4G and Figure S7E).

Taken together, these results indicate that loss of GCS expression in the Arc is significantly involved in part of the metabolic deregulation seen in *Ugcg* ^f/f//CamKCreERT2^ mice.

### GCS-Derived Gangliosides Regulate Leptin Receptor Signaling in Hypothalamic Neurons at the Plasma Membrane

**Figure 5:**
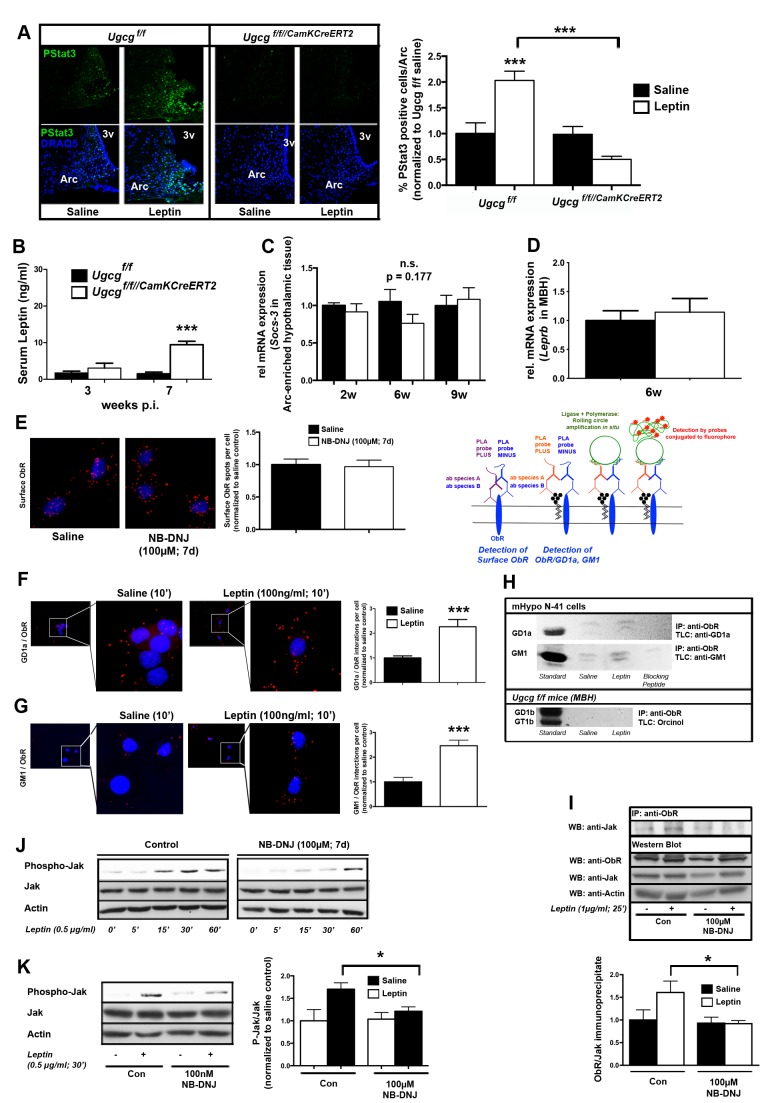
GCS in hypothalamic neurons regulates neuronal leptin signaling at the plasma membrane. (A) Stat3 phosphorylation was markedly decreased in Arc sections of *Ugcg* ^f/f//CamKCreERT2^ mice in response to peripheral leptin (5 mg/kg; 45 min) 6 wk p.i. (*n* = 16–33). Three independent animal groups were analyzed. (B) Serum leptin levels were unchanged 3 wk p.i. and increased prominently 7 wk p.i. in *Ugcg* ^f/f//CamKCreERT2^ mice, reflecting increased body fat mass (*n* = 12–14). (C) mRNA expression analysis for suppressor of cytokine signaling 3 (SOCS-3) expression in Arc-enriched hypothalamic tissue was carried out 2, 6, and 9 wk p.i. *Socs-3* expression normalized to the housekeeping gene tubulin was unaltered (*n* = 3–5). (D) mRNA expression analysis for the long form of the leptin receptor, *Leprb*, in mediobasal hypothalamus was carried out 6 wk p.i. *Leprb* expression normalized to the housekeeping gene tubulin was unaltered at that time point (*n* = 4–5). (E) Immortalized mouse hypothalamic cells (N-41 cells) were analyzed for cell surface expression of ObR. Non-detergent-treated cells were fixed and simultaneously stained with two ObR antibodies. A proximity ligation assay (PLA) indicated quantifiable and unchanged ObR expression on the surface of controls and cells treated with the specific GCS inhibitor NB-DNJ (*n* = 41–47 cells). PLA principle is depicted on the right side. (F–G) N-41 cells were incubated with either saline or 100 ng/ml leptin (10 min). Close interactions between GCS-derived neuronal gangliosides GD1a/ObR (F) and GM1/ObR (G) were detected by PLA. Leptin treatment dynamically increased the GD1a/ObR and GM1/ObR PLA spots per cell (*n* = 48–67 cells). (H) Extracts from saline- and leptin-treated N-41 cells were immunoprecipitated with an ObR antibody, lipids were extracted, and GD1a and GM1 were visualized by immune overlay TLC. GD1a and GM1 co-immunoprecipitated (Co-IP) with ObR, which tended to be stronger in leptin-treated cells. Addition of a blocking peptide almost totally abolished ganglioside signals. Gangliosides GD1b and GT1b, expressed in mouse brain tissue, were not co-precipitated with ObR from hypothalamic tissue of *Ugcg* ^f/f^ mice (5 mg/kg leptin, 45 min). (I) Co-IP showed significantly decreased leptin-induced complex formation between ObR and Jak in NB-DNJ-treated N-41 cells (*n* = 4). (J) Sustainable Jak phosphorylation could be induced in N-41 cells after 15 min of leptin treatment (0.5 µg/ml). NB-DNJ-treated cells showed a markedly delayed response to leptin. (K) Thirty minutes after leptin treatment, Jak phosphorylation was decreased in NB-DNJ-treated cells (*n* = 4). *p≤0.05; ***p≤0.001. Means ± SEM.

Since the number of neurons in the Arc did not differ between *Ugcg* ^f/f//CamKCreERT2^ mice and controls (Figure S8A), a functional analysis of the Arc was performed. Leptin signaling in the hypothalamus is crucial for the maintenance of body weight and energy homeostasis. As adipocyte-secreted leptin is a major regulator of body weight in the CNS, we hypothesized that leptin signaling might be disturbed in GCS-deficient neurons of *Ugcg* ^f/f//CamKCreERT2^ mice. In order to test this hypothesis, we investigated hypothalamic Stat3 phosphorylation (PStat3) in the Arc after peripheral leptin stimulation. Decreased PStat3 was detected by immunofluorescence in the Arc (Figure 5A) and by Western blot in mediobasal hypothalamus (Figure S8B). Interestingly, baseline Stat3 levels were elevated in *Ugcg* ^f/f//CamKCreERT2^ mice (Figure S8C). The PStat3/Stat3 ratio was decreased both at baseline and upon leptin challenge (Figure S8D).

It has been shown that deficient ObR signaling due to leptin resistance of the Arc in mice with diet-induced obesity (DIO) is a consequence of long-term elevated leptin levels [25]–[27]. The suppressor of cytokine signaling 3 (SOCS-3) is a major negative regulator of the ObR that is elevated in rodent models of leptin resistance [25],[28]. In line with progressive obesity, *Ugcg* ^f/f//CamKCreERT2^ mice show indeed elevated leptin levels 7 wk p.i. (Figure 5B). However, expression of hypothalamic *Socs-3* did not rise with increasing obesity and leptin levels, as measured 2, 6, and 9 wk p.i. (Figure 5C). Moreover, hypothalamic ObR expression, usually elevated in leptin-resistant rodents [29],[30], was normal in *Ugcg* ^f/f//CamKCreERT2^ mice 6 wk p.i. (Figure 5D).

To further investigate if GCS-derived gangliosides regulate proper leptin receptor signaling at the level of the plasma membrane in hypothalamic neurons, we first assured that loss of gangliosides would not interfere with ObR transport to the membrane, which would have impaired ObR signaling per se. ObR was labeled by an in situ proximity ligation assay (PLA) on non-detergent-perturbed cells by two ObR antibodies. The number of detected surface ObR PLA spots on cells treated with NB-DNJ was similar to control cells (Figure 5E), indicating that ObR at the plasma membrane of ganglioside-depleted hypothalamic cells is not significantly changed compared to control cells.

As GCS-derived gangliosides have previously been shown to modulate the activity of plasma-membrane-located receptors through close interactions in both adipocytes [17] and neurons [31], we investigated ObR interactions with major neuronal gangliosides. The PLA indicating close proximity events [32] indeed revealed proximity between ObR and gangliosides GM1 and GD1a. In demonstration of activity-dependent interaction between GSL and ObR, the number of GD1a/ObR and GM1/ObR PLA spots increased upon stimulation with leptin (Figures 5F,G and S8E). Complex formation between GD1a/GM1 with ObR was further corroborated by co-immunoprecipitation (Co-IP) of ObR and GD1a/GM1 in saline- and leptin-stimulated N-41 cells (Figures 5H and S8F). As N-41 cells do not express the complex neuronal gangliosides GD1b and GT1b, potential interactions with ObR had to be analyzed in hypothalamic tissue of *Ugcg* ^f/f^ mice. GD1b and GT1b could not be co-precipitated with ObR (Figure 5H).

Ganglioside-depleted cells were then assessed for leptin-dependent signal transduction. Ganglioside-depleted cells did not show the leptin-stimulated increased complex formation between ObR and Jak (Figures 5I and S8G). Time- and dose-dependent Jak phosphorylation could be induced by leptin treatment in N-41 cells and was decreased in NB-DNJ-treated GSL-depleted cells (Figures 5J and S8H). It has to be noted that NB-DNJ evokes ganglioside depletion by only approximately 80%–90% (Figure S3C). Thus, residual gangliosides in the plasma cell membrane may explain the appearance of a P-Jak signal at a late time point after stimulation of NB-DNJ-treated cells. Ganglioside-depleted N-41 cells showed decreased Jak phosphorylation 30 min after stimulation with 0.5 µg/ml leptin (Figure 5J,K).

These results have now shown that two major neuronal GCS-derived gangliosides, GD1a and GM1, form dynamically leptin-stimulated complexes with ObR on the plasma membrane and that loss of gangliosides decreases signal transduction in hypothalamic neurons.

### Distinct Hypothalamic Neurons of *Ugcg* ^f/f//CamKCreERT2^ Mice Are Less Responsive to Peripheral Leptin

**Figure 6:**
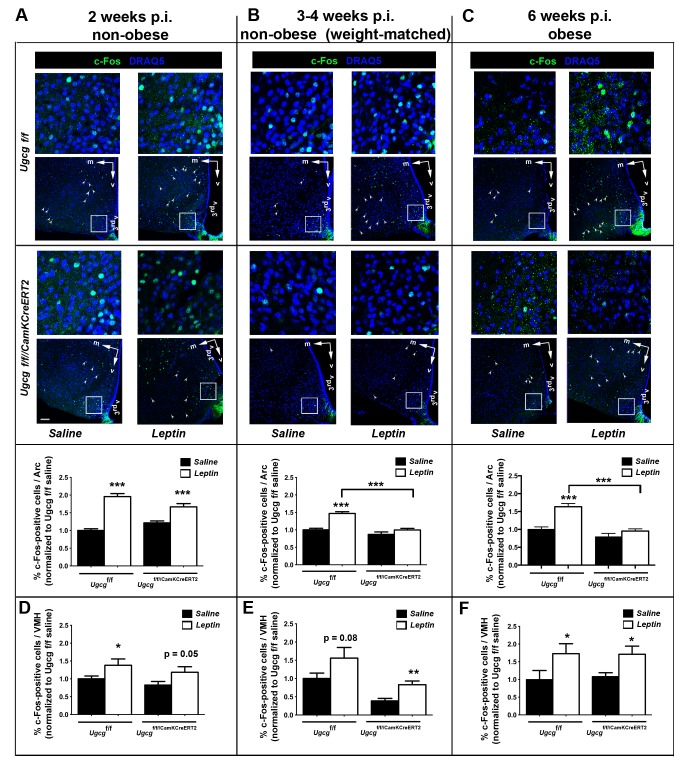
Hypothalamic neurons of *Ugcg* ^f/f//CamKCreERT2^ mice are less responsive to peripheral leptin. (A–C) Brains of leptin-stimulated mice were analyzed for neuronal activity indicated by c-Fos immunofluorescence. Detailed pictures in the upper lane indicate regions of the Arc that are outlined in overview pictures (frames). Arrowheads mark c-Fos-positive neurons located in the VMH. Axis indicators were included indicating the medial (m) and ventral (v) axes. (A) *Ugcg* ^f/f//CamKCreERT2^ mice showed leptin-induced neuronal activation comparable to *Ugcg* ^f/f^ mice in the Arc 1–2 wk p.i. (B) Leptin response in the Arc was decreased in nonobese *Ugcg* ^f/f//CamKCreERT2^ mice weight-matched to controls 3–4 weeks p.i. (C) Decreased c-Fos staining in the Arc was also observed in obese leptin-induced *Ugcg* ^f/f//CamKCreERT2^ mice 6 wk p.i. The percentage of c-Fos-positive neurons per Arc section was depicted as values normalized to saline-injected *Ugcg* ^f/f^ mice (*n* = 14–22 sections). Depicted sections are located between bregma levels −1.5 to −1.8. Quantification contains data from bregma levels −1.4 to −2.3. (D–F) *Ugcg* ^f/f//CamKCreERT2^ mice retained leptin responsiveness in the VMH, as elevated c-Fos after leptin stimulation indicated (*n* = 8–20 sections). Quantification contains data from bregma levels −1.4 to −2.0. Datasets for each time point were acquired individually. Two (1–2 and 3–4 wk) or three (6 wk) independent animal groups were analyzed. Immunofluorescence and image acquisition for each dataset (treated and untreated controls and knockouts) were performed simultaneously. Scale bar: 75 µm; 3^rd^v, 3^rd^ ventricle; *p≤0.05; **p≤0.01; ***p≤0.001. Means ± SEM.

It is known that mice with deficient leptin receptor (db/db mice) function develop obesity and lack hypothalamic responsiveness to leptin stimulation [33]. Regarding the finding that neuronal gangliosides enhance ObR signaling, we hypothesized that hypothalamic neuronal function may be altered in *Ugcg* ^f/f//CamKCreERT2^ mice. In order to investigate this question, neuronal activity after intraperitoneal (i.p.) leptin injection was evaluated by c-Fos staining [34]. Leptin-induced c-Fos formation was normal in non-obese *Ugcg* ^f/f//CamKCreERT2^ mice 1–2 wk p.i. (Figure 6A). Since ganglioside depletion coincides with the start of the obesity development, *Ugcg* ^f/f//CamKCreERT2^ mice that were weight-matched to control littermates were analyzed 3–4 wk p.i. Decreased leptin responsiveness could already be observed in the Arc of these mice (Figure 6B) as well as in the Arc of obese mice 6 wk p.i. (Figure 6C).

Neurons in the nontargeted and non-ganglioside-depleted VMH retained responsiveness to leptin at all time points (Figure 6D–F). As expected, the nontargeted brain stem NTS of *Ugcg* ^f/f//CamKCreERT2^ mice showed regular leptin-induced c-Fos staining 6 wk p.i. (Figure S9).

Altogether, these results indicate a primary deficiency of ganglioside-depleted hypothalamic neurons to respond adequately to peripheral leptin signals.

### Ganglioside-Depleted NPY/AgRP and POMC Neurons in the Arc Are Less Responsive to Leptin

**Figure 7:**
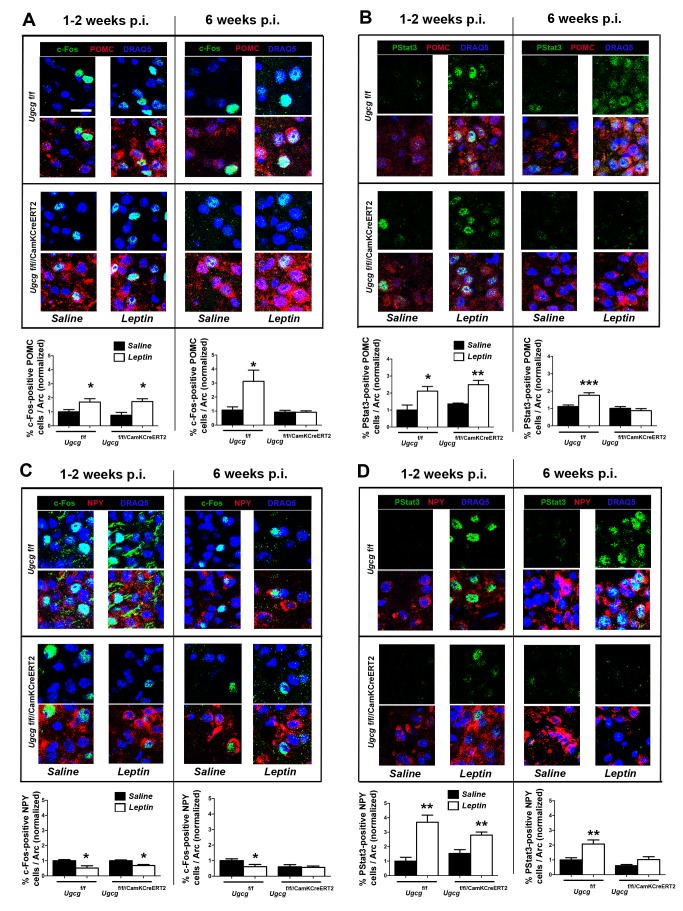
POMC and NPY neurons of *Ugcg* ^f/f//CamKCreERT2^ mice are less responsive to leptin. (A) Leptin engages POMC neurons in the Arc of control (*Ugcg* ^f/f^) mice and *Ugcg* ^f/f//CamKCreERT2^ mice 1–2 wk p.i., as indicated by elevated c-Fos. This response was decreased in Ugcg^f/f//CamKCreERT2^ mice 6 wk p.i. (B) Elevated leptin-induced PStat3 levels in POMC neurons of *Ugcg* ^f/f^ mice and *Ugcg* ^f/f///CamKCreERT2^ mice 1–2 wk p.i. This response was blunted in Ugcg^f/f//CamKCreERT2^ mice 6 wk p.i. (C) Leptin slightly decreased the activity of NPY neurons in *Ugcg* ^f/f^ mice and *Ugcg* ^f/f///CamKCreERT2^ mice 1–2 wk p.i. This was not detected in *Ugcg* ^f/f///CamKCreERT2^ mice 6 wk p.i. (D) Unlike 1–2 wk p.i., leptin did not elevate PStat3 in NPY neurons of *Ugcg* ^f/f///CamKCreERT2^ mice 6 wk p.i. Datasets for each time point were acquired individually, and quantification contains normalized data from two (1–2 wk p.i.; *n* = 4–11) or three (6 wk p.i.; *n* = 18–27) independent animal groups. Immunofluorescence and image acquisition for each dataset (treated and untreated controls and knockouts) were performed simultaneously. Scale bar: 20 µm; *p≤0.05; **p≤0.01; ***p≤0.001. Means ± SEM.

Antagonistic orexigenic NPY and anorexigenic POMC neurons in the hypothalamic Arc are first-order responsive neurons initiating metabolic adaptations to altered peripheral leptin levels [4]. In order to determine leptin-dependent NPY and POMC neuronal function, neuronal activity and ObR activation were assessed by semiquantitative analysis of c-Fos, PStat3, and PIP3 formation in response to peripheral leptin injections. Leptin engaged POMC neurons (α-MSH positive) in control mice, as indicated by increased c-Fos (Figure 7A). Significantly elevated PStat3 (Figure 7B) and PIP3 formation (Figure S10A) confirmed activation of their ObR. Before ganglioside depletion (1–2 wk p.i.), POMC neurons of *Ugcg* ^f/f//CamKCreERT2^ mice responded normally to leptin. However, c-Fos, PStat3, and PIP3 formation were not elevated in response to leptin in obese GSL-deficient mice 6 wk p.i. (Figures 7A,B and S10A). No significant changes were found in mediobasal hypothalamus (MBH) baseline mRNA expression of *Pomc* and cocaine- and amphetamine-regulated transcript (*Cart*) mRNA 6 wk p.i. (Figure S10B).

While a slight decrease in c-Fos–positive NPY neurons was found in leptin-injected control mice, leptin did not show any such effect in *Ugcg* ^f/f//CamKCreERT2^ mice 6 wk p.i. (Figure 7C). Similarly, leptin did not raise PStat3 levels in NPY neurons of *Ugcg* ^f/f//CamKCreERT2^ mice 6 wk p.i. (Figure 7D) and did not have any direct effect on PIP3 formation (Figure S10C). Remarkably, basal mRNA expression of *Agrp* and *Npy* was markedly elevated in the MBH of Ugcg^f/f//CamKCreERT2^ mice 6 and 9 wk p.i., with *Agrp* already increasing 2 wk p.i. (Figure S10D).

**Figure 8:**
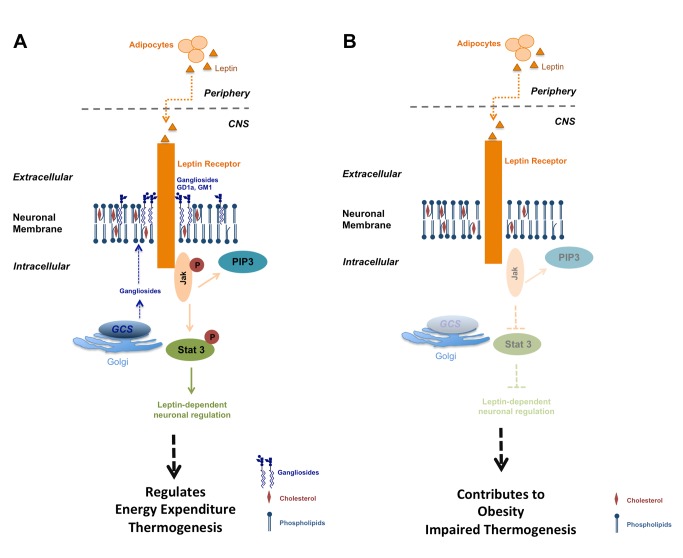
Proposed model for GCS-derived ganglioside GD1a and GM1 regulation of hypothalamic leptin signaling and energy homeostasis. (A) GCS-derived gangliosides form complexes with ObR, thereby facilitating leptin-dependent Jak and Stat3 phosphorylation, and formation of PIP3. These pathways are crucial contributors to regulation of energy homeostasis. (B) In obese *Ugcg* ^f/f//CamKCreERT2^ mice, ObR signal transduction is abolished in GCS-depleted neurons.

In summary, this study has indicated that GCS expression and sufficient gangliosides in neurons of the adult CNS play a seminal role in the regulation of body weight and energy homeostasis. Analysis of the leptin receptor signaling pathway, being one of the most prominent regulators of CNS metabolic control [35],[36], revealed that GCS-derived gangliosides interact with ObR on the plasma cell membrane, thereby facilitating ObR-dependent signal transduction (Figure 8A). In *Ugcg* ^f/f//CamKCreERT2^ mice, leptin responsiveness and neuronal function are impaired in hypothalamic neurons involved in the regulation of energy metabolism (Figure 8B). Consequently, defective ObR signaling contributes to the observed metabolic imbalance and obesity development of mice with ganglioside deficiency in the CNS.

## Discussion

Although the seminal role of CNS feedback responses to peripheral energy signals for the regulation of energy homeostasis has been extensively studied, the role of the lipid microenvironment for energy signal receptor function has not yet been addressed. The present study demonstrates that GCS-derived GSLs are critically involved in a to-date unknown mechanism of hypothalamic control of body weight. In line with the finding that neurons of the constitutive *Ugcg* ^f/f//NesCre^ mice do not show increased apoptosis [15], ganglioside-deficient hypothalamic neurons are viable and they show normal membrane and organelle appearance both in vivo and in vitro. Electrophysiological recordings from Arc neurons in slices of *Ugcg* ^f/f//CamKCreERT2^ mice at 12 wk p.i. did not show a major disruption of membrane functions. However, resting membrane potential and action potential threshold were both shifted to slightly more depolarized values. The molecular mechanism underlying the altered membrane potential remains presently elusive. However, it is well feasible that the shift of threshold is secondary to the slight depolarization, which might inactivate a fraction of Na^+^ channels. In line with the largely normal properties of neurons from brain slices, biophysical parameters of primary hypothalamic neurons devoid of gangliosides were unaltered. Thus, failure of basic electrophysiological membrane functions is unlikely to cause the observed phenotype of mice with ganglioside deficiency.

Therefore, the present work focuses on interactions of leptin receptors with the ganglioside-containing lipid microevironment in which receptors are embedded. We show with independent methods that two major neuronal GCS-derived gangliosides, GD1a and GM1, closely interact with leptin receptors on the neuronal membrane. This interaction is dynamically enhanced by stimulation with leptin. Both *Ugcg* ^f/f//CamKCreERT2^ mice and ganglioside-depleted hypothalamic cells display deficient ObR signal transduction upon leptin stimulation, as assessed by decreased leptin-induced Jak phosphorylation, Stat3 phosphorylation, and PIP3 formation. Corroborated in situ by deficient leptin responsiveness in Arc neurons of *Ugcg* ^f/f//CamKCreERT2^ mice, these results indicate that GCS-derived GSLs, primarily gangliosides, are seminal regulators for neuronal leptin signal transduction. Consequently, *Ugcg* ^f/f//CamKCreERT2^ mice with deficient leptin-induced hypothalamic neuronal responsiveness develop progressive obesity.

Numerous hypothalamic feedback systems involved in body weight maintenance are known [1],[4]. Admittedly, the robust phenotype of *Ugcg* ^f/f//CamKCreERT2^ mice may be caused by several peripheral hormones and defective ensuing signaling events occurring in various Cre-targeted CNS regions of this mouse model. The brain stem NTS, though an important mediator of metabolic control [37], is not targeted by activity under the CamK II *alpha*-dependent Cre recombinase used in this study. Consequently, the NTS shows normal responsiveness to leptin in obese *Ugcg* ^f/f//CamKCreERT2^ mice and can be excluded to contribute to the observed phenotype. Recent reviews also highlight the LHA as an important regulator of energy balance [38],[39]. In fact, compensating neurocircuits involving nontargeted CNS regions may be considered for the return of food intake from initial hyperphagia to normal levels in obese mice despite the striking increase in orexigenic neuropeptides. Even though X-Gal staining could be seen in parts of the LHA, we could not verify Cre targeting of a major part of ObR-expressing LHA neurons in *Ugcg* ^f/f//CamKCreERT2^ mice (Figure S7B). In strong support to this line of reasoning, we demonstrate that partial *Ugcg* replenishment in the Arc mediated by stereotactic injection of rAAV significantly ameliorates obesity and hyperleptinemia in *Ugcg* ^f/f//CamKCreERT2^ mice. Even though limited infection of closely attached tissue by rAAV injection could not be definitely excluded, mainly Arc neurons were targeted by this approach, as assessed by X-Gal stainings of brains co-injected with LacZ-expressing viruses. The present investigation has thus been restricted to GCS effects focused on the MBH harboring Arc neurons.

Deficient leptin signaling as a consequence of leptin resistance occurs predominantly in the Arc of DIO mice with severe long-term hyperleptimenia [25]–[27],[40]. Socs-3 is a major negative feedback pathway of ObR signaling [41]. Thus, elevated Socs-3 expression levels are found in the hypothalamus of leptin-resistant rodent models [25],[42]. In line with observations in obese *db/db* mice with nonfunctioning ObR [25],[42], *Socs-3* expression in the Arc remains indistinguishable from control littermates in nonobese and obese *Ugcg* ^f/f//CamKCreERT2^ mice 2, 6, and 12 wk p.i. Elevated hypothalamic ObR expression, as it occurs in DIO mice [29],[30], has also been proposed as a potential mechanism playing a role in the development of leptin resistance [28]. However, normal ObR expression in *Ugcg* ^f/f//CamKCreERT2^ mice supports the hypothesis that the ObR signaling in their neurons must be deficient due to ganglioside loss and not merely due to secondary leptin resistance. Furthermore, the nontargeted hypothalamic VMH and brain stem NTS retain leptin responsiveness even in obese mice 6 wk p.i. These results in combination with the decreased ObR signal transduction in ganglioside-depleted and non-leptin-resistant N-41 cells strongly suggest that loss of GCS-derived GSLs including gangliosides GD1a and GM1 is the reason for failing ObR activation and subsequently inhibited intracellular signaling.

GCS-depleted Arc neurons display normal leptin sensitivity 1–2 wk p.i., a time point when gangliosides are still present. Furthermore, onset of body weight gain, deficient neuronal activity in the Arc, and abolished ObR signaling coincide with ganglioside depletion 3 wk p.i. This strongly suggests that the mentioned defects are due to ganglioside depletion in these cells rather than due to lack of the enzyme GCS itself.

Further evidence for the postulate that ganglioside deficiency-dependent inhibition of ObR signaling in hypothalamic neurons leads to impaired neuronal function is based on our in situ results in ganglioside-depleted Arc of both obese and nonobese *Ugcg* ^f/f//CamKCreERT2^ mice. Whereas leptin injection increases c-Fos immunoreactivity and thus neuronal activity in the Arc neurons of fasted lean mice, this response did not occur in GCS-deficient neurons. Leptin specifically engages POMC neurons. Even though the effects of PI3k- and Stat3-dependent signaling in POMC neurons do not overlap [12],[43], both pathways are activated by leptin [11],[12],[44],[45] and contribute to maintenance of energy homeostasis [46]. In ganglioside-depleted POMC neurons, neither PStat3 nor PIP3 formation is increased by peripheral leptin injections, strongly suggesting that defects in both pathways may contribute to partial failure of obesity prevention. As peripheral leptin stimulates both pathways through ObR activation [11],[47], defective ObR function is very likely to be assumed.

In NPY neurons, it has been demonstrated that Jak-Stat3 signaling plays an important role in maintaining NPY/AgRP-mediated energy homeostasis [48]. Additional ObR-mediated PI3k activation seems to be required for inhibiting *Npy* and *Agrp* gene expression [8]. *Npy* and *Agrp* expression is markedly increased in the MBH of *Ugcg* ^f/f//CamKCreERT2^ mice, which may be a consequence of absent leptin-induced PStat3 formation in NPY neurons. On the other hand, leptin-induced PIP3 formation does not differ in neither of the groups, which goes in line with the hypothesis that leptin-dependent PIP3 formation in AgRP/NPY neurons is stimulated by an indirect mechanism involving synaptic transmission [12]. Overactive NPY neurons in obese ObR-deficient *Lepr* ^fa/fa^ rats were shown to inhibit sympathetic nervous outflow to BAT and cause hypothermia [7] as observed in *Ugcg* ^f/f//CamKCreERT2^ mice. With regard to the fact that *Npy* and *Agrp* but not *Pomc* expression differ in *Ugcg* ^f/f//CamKCreERT2^ mice, the role of GCS expression in regulating neuropeptide expression and secretion has to be elucidated. Especially the role of hypothalamic insulin receptor signaling, which also regulates the expression of *Pomc* and *Npy/Agrp* in part similar to ObR signaling [11] and is antagonized by GM3 in the periphery [17], constitutes a promising target for further clarifying the differential neuropeptide expression. Moreover, a potential contribution of Cre-targeted ObR-expressing neurons in the median preoptic area of *Ugcg* ^f/f//CamKCreERT2^ mice to hypothermia may also be considered.

Dynamic membrane microdomains are widely accepted as critical components involved in membrane receptor functions [16],[49]. Since GCS-derived gangliosides are important constituents of these microdomains, they potentially interact with and regulate a variety of membrane components including receptors such as Trk receptors [31] and insulin receptors [17]. In contrast to mice with neuron-specific insulin receptor deletion, which only display a gender- and diet-dependent subtle increase in body weight [11],[50], the obesity and glucose intolerance observed in db/db mice can be rescued by neuron-specific re-expression of ObR [51]. Furthermore, deficient ObR signaling in POMC neurons of the Arc itself leads to the development of mild obesity [52]. In consideration of these findings—despite the existence of potential alternative pathways that might be impaired in neurons of *Ugcg* ^f/f//CamKCreERT2^ mice—we ascribe ObR and its regulation of activity to a major function in our model pointing to a novel mechanism for CNS metabolic regulation.

We demonstrate that GCS-derived gangliosides GD1a and GM1 closely interact with ObR. The leptin-induced increase in GD1a/ObR and GM1/ObR interaction assumes recruitment of these gangliosides to the ObR upon leptin stimulation. These results in combination with the demonstrated deficient ObR signaling in ganglioside-depleted hypothalamic neurons both in vivo and in vitro leads us to surmise that the lipid microenvironment surrounding the ObR can significantly modulate leptin-dependent intracellular signal transduction in hypothalamic neurons. Altogether, these results provide evidence that GM1 and GD1a are actively involved in enhancing the effects of leptin in hypothalamic neurons.

As insulin receptors contain a lysine residue predicted for interaction with GM3 [53], loss of GM3 synthase showed already a prominent effect on peripheral insulin receptor signaling [17]. It is a widely accepted concept that in the state of insulin resistance in peripheral adipocytes, the IR segregates from caveolae into GM3-enriched microdomains [53], an endogenous inhibitory mechanism [17]. Indeed, elevated GM3 synthase expression could be detected in adipose tissue of obese Zucker *fa/fa* rats and *ob/ob* mice [54]. Pharmacologic GCS inhibition in the periphery has been shown to exert beneficial effects on peripheral insulin sensitivity and liver steatosis [55],[56]. With regard to the fact that different ganglioside species can exert either stimulatory [18],[57] or inhibitory [17] effects on membrane receptors, the mentioned studies including the present work support the concept that any perturbation, either loss or excess, of membrane GSLs can alter receptor function. Contributions of GCS-derived lipid raft components apart from gangliosides, namely neutral GSLs in the CNS, to leptin receptor function have yet to be elucidated and constitute a challenging target for future investigations. Besides gangliosides, lactosylceramide has been shown to contribute to formation of lipid microdomains [58]. We, however, propose in the present study that in line with the findings for the insulin receptor, hypothalamic leptin receptor signaling is to a significant extent regulated through interactions with the dominant gangliosides GD1a and GM1.

Recent studies have highlighted the central role of systemic ceramide biosynthesis and GCS in the regulation of energy homeostasis [59],[60]. In accordance with earlier findings [15],[61], we show that neuronal ceramide levels in *Ugcg* ^f/f//CamKCreERT2^ mice are indistinguishable from control mice, virtually excluding any effects of ceramides.

In conclusion, our study highlights the expression of neuronal GCS-derived GSLs, foremost gangliosides, as a novel class of hypothalamic metabolic regulators. Gangliosides GM1 and GD1a enhance the action of leptin on intracellular signaling and neuronal activity, most likely through dynamic changes of the lipid microenvironment of the ObR. We demonstrate by independent methods that gangliosides GD1a and GM1 strongly interact with the ObR upon leptin stimulation. Loss of these gangliosides leads to impaired responsiveness. By this relevant influence on hormone signaling, *Ugcg* deletion in adult mouse CNS leads to development of progressive obesity, hyperleptinemia, and glucose intolerance. The obesity can be partially ameliorated by restoration of GCS activity and ganglioside expression in the hypothalamic Arc of *Ugcg* ^f/f//CamKCreERT2^ mice. Neuronal GCS expression therefore constitutes a novel mechanism for hypothalamic regulation of body weight maintenance.

## Materials and Methods

### *Ugcg* ^f/f//CamKCreERT2^ Mice

Animals were kept in specific-pathogen-free barrier facilities. *Ugcg* ^f/f^ mice [15] and inducible CamKCreERT2 mice were bred to generate *Ugcg* ^f/f//CamKCreERT2^ mice and control littermates. Mice were induced with tamoxifen 6 wk after birth for 1 wk as described [19]. We performed experiments in female mice, unless stated otherwise.

### Glucose Tolerance and Insulin Sensitivity

Mice were fasted overnight (o/n). Blood glucose levels were analyzed prior to i.p. injection of glucose (2 g/kg body weight). Glucose levels were determined from tail vein blood (Glucometer Accu Check, Aviva, Roche). Food was withdrawn 4 h prior to the insulin sensitivity assay. Mice were injected i.p. with 0.75 U/kg human insulin (Eli Lilly), and glucose levels were determined as described above (see also Text S1).

### GD1a Immunofluorescence

Staining was carried out as described earlier [62]. Cryosections (male mice) were incubated with mouse-α-GD1a (1∶100, Millipore) followed by secondary donkey-α-mouse-Alexa-Fluor 488 (1∶200, Invitrogen). Analysis was performed by confocal microscopy (TCS-SL, Leica).

### Leptin Injections and Double-Immunofluorescence

Mice were fasted o/n and injected with leptin (5 mg/kg, Peprotech) or saline between 8.00 a.m. and 10.00 a.m. Animals were sacrificed at indicated time points and transcardially perfused with 4% paraformaldehyde (PFA). We prepared 40 µm cryosections covering the Arc. Alternating sections were collected in series for subsequent free-floating section immunostainings. First antibodies used for immunostaining were rabbit-α-PStat3 (1∶100, Cell Signaling Technology), rabbit-α-c-Fos (1∶100, Santa Cruz), and FITC-conjugated α-PIP3 (1∶100, Echelon). Secondary antibody was donkey-α-rabbit-Alexa-Fluor 488 (1∶200, Invitrogen). Sections were subsequently incubated with either goat-α-NPY (1∶50, Santa Cruz) or sheep-α-MSH (1∶1000, Millipore) followed by secondary antibodies (donkey-α-goat-Alexa-Fluor 546, donkey-α-sheep-Alexa-Fluor 546, 1∶200, Invitrogen). Stainings were analyzed by confocal microscopy (TCS-SL, Leica). Neurons with nuclear (c-Fos, PStat3) or cytoplasmic (PIP3, NPY, and α-MSH) staining above background were considered positive. Immunofluorescence for beta galactosidase was performed as described earlier [63].

### Generation of rAAV

The AAV Helper-free System (Agilent Technologies Inc.) was used for preparation of rAAV. Full-length mouse *Ugcg* cDNA was cloned into the pAAV-MCS vector from the Helper-free System (pAAV-Ugcg). Viruses were generated according to the manufacturer's guidelines and purified as described earlier [64].

### rAAV Injection Into the Arc

Bilateral stereotaxic injections were performed as described [65]. We injected 400 nl virus solution containing equal volumes of viruses carrying *Ugcg* and *lacZ* (∼1.8*10^11^ genome copies/ml) into the Arc of each hemisphere (caudal to bregma: 1.4 mm, 1.44 mm; lateral: 0.25 mm; ventral: 5.7 mm). After surgery, mice were maintained with *ad libitum* access to lab chow and body weight was monitored weekly.

### Immortalized Hypothalamic Cell Culture (N-41cells)

Immortalized hypothalamic neurons were purchased from CELLutions Biosystems (mHypoE N-41, Cedarlane) and cultured according to the manufacturer's guidelines. GCS was inhibited with NB-DNJ treatment (100 µM, 7 d, Sigma; Tocris).

### Proximity Ligation Assay (PLA)

Eight thousand N-41 cells were seeded onto coverslips and incubated at 37° o/n. The 3 h serum-starved cells were stimulated with leptin (100 ng/ml, Peprotech) for 10 min, washed with PBS and fixed in 4% PFA for 15 min. Cells were blocked with 5% skim milk/PBS. PLA was performed with primary antibodies against ObR (1∶50, Santa Cruz), GD1a (1∶100, Millipore), and GM1 (1∶10, Matreya). PLA was performed according to the manufacturer's guidelines (Duolink Orange Detection System, Olink Biosciences). Formation of PLA spots was analyzed by fluorescence microscopy (Zeiss Cell Observer).

### Western Blot

Mice were injected with leptin or saline as described above and sacrificed 30 min later. The MBH was dissected homogenized on ice in lysis buffer (20 mM HEPES, 25 mM KCl, 250 mM sucrose, 2 mM MgCl_2_, 0.5 mM DTT, 1% digitonin) containing proteinase inhibitor (Roche) and phosphatase inhibitor cocktail (Sigma). Immortalized hypothalamic cells were treated with either saline or 100 µM NB-DNJ for 7 d, serum starved for 4 h, and subsequently treated with either saline or leptin (1,000 ng/ml, Peprotech, 1 h). Cells were lysed on ice in lysis buffer. Protein concentrations were determined by Bradford assay (Sigma). Western blots were performed as described earlier [25]. Primary antibodies: rabbit-α-PStat3, rabbit-α-Stat3, rabbit-α-PJak, rabbit-α-Jak (1∶1,000, Cell Signaling Technology), mouse-α-tubulin (1∶5,000, Zymed Labs), and rabbit-α-actin (1∶1,000, Santa Cruz). Secondary antibodies: HRP-conjugated α-rabbit-IgG (1∶1,000, Dako) and HRP-conjugated α-mouse-IgG (1∶5,000, Santa Cruz). Bands were visualized by chemiluminescence (Amersham) and quantified (ImageJ, NIH).

### Co-Immunoprecipitation (Co-IP)

Four hours serum-starved N-41 cells were treated with leptin (1,000 ng/ml, 25 min). Cells were lysed in IP buffer [50 mM HEPES, pH 7.0, 150 mM NaCl, 10% glycerol, 1% Triton-X, 1.5 mM MgCl_2_, 1 mM EDTA, proteinase inhibitor cocktail (Roche)]. Co-IP for ObR/Jak was performed as described earlier [36]. ObR/GD1a- and ObR/GM1-Co-IP and subsequent lipid extraction and analysis was performed as described earlier [31],[53]. Anti-ObR were incubated at 4°C o/n. Immunoprecipitated lipids were desalted on an RP-18 column, spotted on a TLC, and run in solvent (chloroform/methanol/0.2% CaCl_2_; 60∶35∶8, by vol.). GD1a was visualized with mouse α-GD1a (1∶1,000, 4°C overnight, Millipore) on the TLC by immune overlay staining as described earlier [66].

### Measurement of Serum Leptin and Nonesterified Free Fatty Acids (NEFAs)

Serum leptin and NEFAs were determined by commercially available kits according to the manufacturer's guideline [Leptin-ELISA (Linco); NEFA-HR2 kit (WAKO Chemicals)]. NEFAs were measured in male mice.

### Determination of NETO and NE Content in iBAT

NETO rate in iBAT was determined as described earlier [67]. Tissue NE was measured by reversed-phase HPLC with electrochemical detection (Chrome Systems, Germany) (see also Text S1).

### Metabolic Characterization, Core Body Temperature, and Locomotor Activity

Body weight was measured once a week. Metabolic measurements were carried out in an open circuit respiratory system (SM-MARS, Sable Systems, USA). VO_2_ and VCO_2_ per mouse were analyzed for 21 h to determine the RER = V_CO2_/V_O2_ and HP (mW). Whole body composition was determined by noninvasive NMR analysis (Mini-Spec, Bruker Optics). Core body temperature was measured with a rectal probe (ALMENO 2390-1, Ahlborn) (see also Text S1).

### Quantitative mRNA Analysis

Total RNA of the MBH was extracted from nonfasted mice as described earlier [68]. RNA was reversely transcribed by Superscript II Reverse Transcriptase (Invitrogen) and cDNA was quantified using the LC FastStart DNA Master SYBR Green I kit (Roche) according to the manufacturer's guidelines and the Light Cycler (Roche) (see also Text S1).

### X-Gal Stainings

*R26R/Ugcg* ^f/+//CamKCreERT2^ mice and *R26R/Ugcg* ^f/+^ mice were induced with tamoxifen i.p. 6 wk after birth as described. At 3 d p.i., animals were sacrificed, and brains were removed and frozen on dry ice. X-Gal staining was performed as described previously [69]. Similarly, β-galactosidase activity in brains of rAAV-Ugcg/LacZ-, rAAV-Empty/LacZ-, and rAAV-LacZ-injected mice was visualized 7 d after virus injection.

### Extraction and Analysis of GSLs and Ceramide

GSLs were extracted and separated into neutral and acidic fractions containing gangliosides as described earlier [15]. The amount of GSLs spotted onto a plate by a TLC applicator (Camag, USA) was normalized to tissue protein content determined by the Lowry method [70]. TLC running solvent for acidic GSL was chloroform/methanol/0.2% CaCl_2_ (45∶45∶10 by vol). GSLs were visualized with 0.2% orcinol in 10% sulphuric acid at 120°C for 10 min. Ceramide was extracted as described earlier [15] and spotted onto a TLC plate. Running solvent for ceramide was chloroform/methanol/acetic acid (190∶9∶1 by vol), and ceramide was visualized with 10% CuSO_4_ in 8% H_3_PO_4_ at 180°C for 10 min. Lipid content was quantified by densitometry (Shimadzu, Japan).

### Statistical Analysis

Unless stated elsewhere, results were analyzed by a two-tailed, unpaired Student's *t* test (Graph Pad Prism, Graph Pad Software, Inc.). To analyze main effects of genotype on metabolizable energy or energy expenditure, body weight was employed as a co-factor in a linear regression model to account for the confounding effect of body size on energy metabolism parameters [71]. *p*≤0.05 was considered statistically significant and marked *. *p*≤0.01 was marked **, and *p*≤0.001 was marked ***.

## Supporting Information

Figure S1 Generation and characterization of inducible *Ugcg* ^f/f//CamKCreERT2^ mice. (A) *R26R/Ugcg* ^f/+//CamKCreERT2^ reporter mice and *R26R/Ugcg* ^f/+^ mice were induced with tamoxifen and brains were removed 3 d after the last injection. X-Gal staining revealed Cre activity in hypothalamic MnPO, paraventricular nucleus (PVN), and lateral hypothalamus (LH). Cre activity was absent in ventromedial hypothalamus (VMH) and in the brain stem nucleus of the solitary tract (NTS; cc, central canal). Cre activity could be detected in hippocampus and cerebral cortex. GD1a immunofluorescence visualized ganglioside depletion in Cre-targeted regions of male *Ugcg* ^f/f//CamKCreERT2^ mice (6 wk p.i). Scale bar: 100 µm. (B) Overview of brain slices from *R26R/Ugcg* ^f/+//CamKCreERT2^ reporter mice indicating Cre activity. 3v, 3^rd^ ventricle; CP, caudoputamen; LSN, lateral septal nucleus; SFO, subfornical organ; CTX, cortex; HC, hippocampus; DMH, dorsomedial hypothalamus. (C) In situ hybridization showed depletion of *Ugcg* mRNA in hypothalamic PVN (a), SCN (b), as well as in the amygdala (c), hippocampus (d), and cerebral cortex (e) of male *Ugcg* ^f/f//CamKCreERT2^ mice (4 wk p.i.). Respective areas were visualized by thionine staining. (D) Southern blot did not show recombination events in peripheral tissues of female *Ugcg* ^f/f//CamKCreERT2^ mice 2 wk p.i. The *Ugcg* null allele could solely be detected in cerebral cortex, hippocampus, and hypothalamus. (E) Stable ganglioside depletion 3 wk p.i. in targeted neuronal populations of totally dissected hippocampus as shown by TLC. Residual gangliosides from nontargeted cells and innervating fibers were still visible. (+ *Ugcg* ^f/f^; − *Ugcg* ^f/f//CamKCre ERT2^). Lipid amounts equaling 50 µg of tissue protein were loaded. Quantification from densitometry analysis of thin layer chromatography results is depicted (*n* = 3). (F) Sphingomyelin was slightly elevated in hippocampus of *Ugcg* ^f/f//CamKCreERT2^ mice. *p≤0.05; **p≤0.01. Means ± SEM.
(TIF)

Figure S2 Intrinsic electrical properties of Arc neurons from brain slices of female *Ugcg* ^f/f//CamKCreERT2^ mice at 12 wk p.i. compared to controls. (A) Spontaneous firing frequency (left), maximal rate of rise of action potentials (AP; middle), and AP width at half-maximal amplitude (right) were not different between both strains. (B) Resting membrane potential (RMP, left) and action potential threshold (right) were both shifted to more depolarized values in cells from *Ugcg* ^f/f//CamKCreERT2^ mice. Cell numbers are stated in brackets above each column. *p≤0.05. Mean values ± SEM.
(TIF)

Figure S3 Immortalized hypothalamic cells (N-41 cells) express gangliosides and ganglioside-depleted cells show normal morphological (N-41) and biophysical membrane properties (primary *Ugcg* ^f/f//NesCre^ neurons). (A) Ganglioside expression pattern as determined by TLC after lipid extraction from immortalized hypothalamic cell lines and subsequent separation of neutral and acidic glycosphingolipids including gangliosides (St, standard). (B) Immune overlay TLC confirmed the identity of GD1a and GM3 bands (St, standard). (C) The 7-d treatment of immortalized hypothalamic cells with the GCS inhibitor NB-DNJ (100 µM) led to inhibition of ganglioside biosynthesis, as shown by TLC (St, standard). Quantification of the TLC bands is depicted. (D) Normal membrane appearance and assessed by electronmicroscopy (n, nucleus; nl, nucleoli; m, mitochondria; er, endoplasmic reticulum; pm, plasma membrane). Scale bar: 2 µm. (E). Basic electrophysiological characteristics of primary *Ugcg* ^f/f^ and *Ugcg* ^f/f//NesCre^ neurons from total embryonic hypothalamus (E16–E18). Whole-cell recordings from randomly picked neurons between 7 DIV and 12 DIV. Membrane resistance and capacitance (tested with a 5 mV test pulse) as well as resting membrane potential were unaltered in *Ugcg* ^f/f//NesCre^ neurons. (F) Action potential threshold, amplitude and duration were also unaltered in *Ugcg* ^f/f//NesCre^ neurons. Data are given as mean values ± SEM.
(TIF)

Figure S4 Fat mass in *Ugcg* ^f/f//CamKCreERT2^ mice was elevated, while organ morphology was unaltered. (A) Unaltered body weight in heterozygous *Ugcg* ^f/+//CamKCreERT2^ mice (*n* = 4). (B) NMR analysis revealed that lean mass was only slightly increased 4 wk p.i. (*n* = 9–10). (C) Significantly increased body fat mass in *Ugcg* ^f/f//CamKCreERT2^ mice 7 and 10 wk p.i. when adjusted for body weight, as determined by nuclear magnetic resonance imaging (*n* = 9–10 per group); **p≤0.01 (LM). Individual values. Relative lean mass, when plotted against body weight, decreased during progressive weight gain 7 and 10 wk p.i. in *Ugcg* ^f/f//CamKCreERT2^ mice (*n* = 9–10 per group); *p≤0.05; ***p≤0.001 (LM). Individual values. (D) Unaltered morphology of major peripheral organs in female mice was shown by hematoxylin and eosin stainings 9 wk p.i. No liver steatosis could be detected. (Scale bars: Liver, 50 µm; Kidney, Lungs, Spleen, 200 µm). (E) Parameters indicative for liver function did not show any significant changes 19 wk p.i., indicating normal liver function in *Ugcg* ^f/f//CamKCreERT2^ mice (*n* = 12; GOT, *n* = 4). (F) Serum levels for cholesterol, fasted glucose, urea, and creatinine 19 wk p.i. did not show any biologically relevant differences in mice (*n* = 12). (G) *Ugcg* ^f/f//CamKCreERT2^ mice displayed impaired glucose tolerance 12 wk p.i. (*n* = 9; Mann-Whitney Rank Sum Test). (H) Slight but significant insulin insensitivity was detected in *Ugcg* ^f/f//CamKCreERT2^ mice 10 wk p.i. (*n* = 8–9; Mann-Whitney Rank Sum Test). *p≤0.05; **p≤0.01; ***p≤0.001. Means ± SEM unless stated otherwise.
(TIF)

Figure S5 Normal excretion and unaltered spontaneous locomotor activity in *Ugcg* ^f/f//CamKCreERT2^ mice, but plasma NEFAs are altered. (A) Fecal free fatty acids (8 wk p.i.), fecal energy content, and assimilation coefficient were not altered in *Ugcg* ^f/f//CamKCreERT2^ mice 3, 6, and 11 wk p.i. Means ± SEM. (B) Metabolic rate of *Ugcg* ^f/f//CamKCreERT2^ mice was unaltered 2 wk p.i. (*n* = 7–10). Individual means. (C) Spontaneous locomotor activity measured at different time points after tamoxifen induction did not reveal any statistically significant difference between *Ugcg* ^f/f//CamKCreERT2^ mice and control littermates (*n* = 9) (Mann-Whitney Rank Sum Test). Medians and individual values. (D) Decreased plasma NEFA values in fasted *Ugcg* ^f/f//CamKCreERT2^ mice 11 wk p.i., indicating decreased fat mobilization (*n* = 4–6). **p≤0.01. Means ± SEM.
(TIF)

Figure S6 Hypothermia in *Ugcg* ^f/f//CamKCreERT2^ mice is not due to defective thyroid function, but decreased sympathetic activity in iBAT. (A) Enlarged lipid droplets were found in iBAT of *Ugcg* ^f/f//CamKCreERT2^ mice 9 wk p.i. (B) Altered mitochondrial density and size were detected in *Ugcg* ^f/f//CamKCreERT2^ mice by ultrastructural analysis of iBAT 9 wk p.i. (C) Mitochondrial area was reduced in *Ugcg* ^f/f//CamKCreERT2^ mice (*n* = 3 mice, 195 mitochondria). (D) Serum-free thyroxine (fT4) was determined by ELISA and did not show any alterations in *Ugcg* ^f/f//CamKCreERT2^ mice (*n* = 5–6 per group). (E) Serum-free triiodothyronine (fT3) was determined by ELISA and did not show any alterations in *Ugcg* ^f/f//CamKCreERT2^ mice (*n* = 3–6 per group). (F) High performance liquid chromatography (HPLC) revealed lower sympathetic activity (NETO rate) per mg iBAT 9 wk p.i. (*n* = 4). (G) NE content was also decreased in *Ugcg* ^f/f//CamKCreERT2^ iBAT 9 wk p.i. (*n* = 4). **p≤0.01; ***p≤0.001. Means ± SEM.
(TIF)

Figure S7 rAAV-mediated *Ugcg* gene delivery to the hypothalamic Arc ameliorates obesity in *Ugcg* ^f/f//CamKCreERT2^ mice. (A and B) Double immunofluorescence showed that Cre activity, indicated by beta galactosidase staining (b-gal), was targeted to MnPO neurons (A) expressing the long form of the ObR (see arrowheads), but not to the majority of ObR neurons in the LHA (B), as indicated by PStat3 staining in leptin-injected *R26R/Ugcg* ^f/+//CamKCreERT2^ mice (5 mg/kg leptin, 120 min). (C) Stereotactic rAAV-LacZ delivery to the Arc was exemplarily demonstrated by X-Gal-staining. Morphology was depicted in the HE section. (D) *Ugcg* ^f/f//CamKCreERT2^ mice that were not targeted in the Arc by stereotactic delivery of rAA viruses encoding *Ugcg* and *lacZ* did not show improvement in body weight increase compared to rAAV-Empty/lacZ-injected *Ugcg* ^f/f//CamKCreERT2^ mice (*n* = 4 rAAV-Ugcg/lacZ missed, *n* = 8 rAAV-Empty/lacZ). The graph depicting rAAV-Empty/lacZ-targeted mice is taken from Figure 4B for comparison. (E) Restored ganglioside biosynthesis in the Arc of rAAV-Ugcg-injected *Ugcg* ^f/f//CamKCreERT2^ mice, as shown by GD1a immunofluorescence 8 wk p.i. Shown are overview pictures for Figure 4G (also taken for Figure S7E). Scale bar: 18 µm. Means ± SEM.
(TIF)

Figure S8 GCS in hypothalamic neurons regulates neuronal leptin signaling. (A) Neuron count in the Arc was normal in *Ugcg* ^f/f//CamKCreERT2^ mice (*n* = 115–122 sections). Quantification contains normalized data from 12 mice each. (B) Stat3 phosphorylation in MBH of male mice was investigated by Western blot 6 wk p.i. We loaded 100 µg of protein in each lane. Elevated PStat3 levels were not seen in *Ugcg* ^f/f//CamKCreERT2^ mice upon leptin stimulation. Quantification for PStat3/Tubulin is depicted (*n* = 3). (C) Baseline and leptin-stimulated Stat3 levels were elevated in *Ugcg* ^f/f//CamKCreERT2^ mice. Stat3 was normalized for tubulin expression (*n* = 3). (D) The PStat3/Stat3 ratio is decreased in *Ugcg* ^f/f//CamKCreERT2^ mice 6 wk p.i. both at baseline and after leptin stimulation. PStat3 and Stat3 levels were normalized for tubulin (*n* = 3). (E) Immortalized hypothalamic cells (N-41 cells) were incubated with either saline or 100 ng/ml leptin (10 min). A proximity ligation assay (PLA; principle depicted in Figure 5E) for GD1a/ObR similar to the experiment depicted in Figure 5F was performed using a different ObR-specific antibody. GD1a/ObR interactions were detected and leptin treatment dynamically increased the GD1a/ObR PLA spots per cell similar to the result depicted in Figure 5F. Pre-adsorption of the antibody by a blocking peptide abolished PLA signals (*n* = 74–150 cells). (F) Similar to the experiments depicted in Figure 5H, extracts from saline- and leptin-treated N-41 cells were immunoprecipitated with a second ObR antibody, lipids were extracted, and GD1a and GM1 were visualized by immune overlay TLC. Similar to the results obtained in Figure 5H, GD1a and GM1 co-immunoprecipitated (Co-IP) with ObR. (G) Similar to the result in Figure 5I, Jak was co-precipitated with the ObR, which increased upon leptin stimulation of N-41 cells, using a second ObR antibody. Addition of the blocking peptide abolished the signal. (H) Weak Jak phosphorylation was induced in N-41 cells after 30 min of leptin treatment (0.1 µg/ml). *p≤0.05; **p≤0.01; ***p≤0.001. Means ± SEM.
(TIF)

Figure S9 The brain stem NTS of *Ugcg* ^f/f//CamKCreERT2^ mice retained leptin responsiveness 6 wk p.i. c-Fos expression was unaltered in the nontargeted NTS (*n* = 2–4 sections). Datasets for each time point were acquired individually. Immunofluorescence and image acquisition for each dataset (treated and untreated controls and knockouts) were performed simultaneously. *p≤0.05; **p≤0.01. Means ± SEM.
(TIF)

Figure S10 Reduced PIP3 formation in POMC neurons of *Ugcg* ^f/f//CamKCreERT2^ mice upon leptin stimulation. (A) Fasted mice were injected with either saline or leptin (5 mg/kg body weight) and sacrificed 45 min later. Either POMC or NPY staining identified individual neuronal populations. Leptin evokes PIP3 formation in Arc POMC neurons of *Ugcg* ^f/f^ mice as well as *Ugcg* ^f/f//CamKCreERT2^ mice before ganglioside depletion was completed (1–2 wk p.i.). This response was blunted in ganglioside-depleted POMC neurons 6 wk p.i. The percentage of POMC/PIP3-double-positive neurons per Arc section normalized to Ugcg^f/f^ saline is depicted (*n* = 3–7). (B) Unaltered mRNA expression of the anorexigenic neuropeptides POMC and CART in the MBH of *Ugcg* ^f/f//CamKCreERT2^ mice 6 and 9 wk p.i. (*n* = 3–6). (C) Fasted mice were injected with either saline or leptin (5 mg/kg body weight) and sacrificed 45 min later. NPY staining identified individual neuronal populations. Leptin did not directly lead to increased PIP3 formation in NPY neurons in neither of the groups (*n* = 3–7). (D) Increased *Npy* and *Agrp* mRNA expression in the MBH of *Ugcg* ^f/f//CamKCreERT2^ mice 6 and 9 wk p.i. (*n* = 3–6). Datasets for each time point were acquired individually. Immunofluorescence and image acquisition for each dataset (treated and untreated controls and knockouts) were performed simultaneously. *n* = 3–7 sections; *p≤0.05; **p≤0.01; ***p≤0.001. Means ± SEM.
(TIF)

Text S1 Supplemental experimental procedures and supplemental references.
(DOC)

## Abbreviations

3v: third ventricle
AgRP: agouti-related peptide
alpha-MSH: alpha-melanocyte stimulating hormone
AP: action potential
Arc: arcuate nucleus
CamKCre: calcium/calmodulin-dependent kinase II alpha Cre
CART: cocaine and amphetamine-regulated transcript
CNS: central nervous system
CP: caudoputamen
d: days
DIO: diet-induced obesity
DMH: dorsomedial hypothalamus
E_MET_: metabolizable energy
FFAs: free fatty acids
GCS: glucosylceramide synthase
GSLs: glycosphingolipids
HPLC: high performance liquid chromatography
iBAT: intrascapular brown adipose tissue
i.p.: intraperitoneal
Jak: janus kinase
LHA: lateral hypothalamic area
MBH: mediobasal hypothalamus
MnPO: median preoptic area
NB-DNJ: n-butyldeoxynojirimycin
NE: norepinephrine
NEFAs: non-esterified free fatty acids
Nes: nestin
NETO: norepinephrine turnover rate
NMR: nuclear magnetic resonance
NPY: neuropeptide Y
NTS: nucleus of the solitary tract
ObR: leptin receptor
p.i.: postinduction
PFA: paraformaldehyde
PI3k: phosphatidylinositol-3-OH kinase
PIP3: phosphatidylinositol(3,4,5)-triphosphate
PLA: proximity ligation assay
POMC: proopiomelanocortin
PVN: paraventricular nucleus
rAAV: recombinant adeno-associated virus
RER: respiratory exchange ratio
RMP: resting membrane potential
SFO: subfornical organ
SOCS-3: suppressor of cytokine signaling 3
Stat: signal transducer and activator of transcription
TLC: thin layer chromatography
Ugcg: UDP-glucose:ceramide glucosyltransferase
VMH: ventromedial hypothalamus
WAT: white adipose tissue
wk: weeks
