# Supplementary figures and images for: Neuronal Expression of Glucosylceramide Synthase in Central Nervous System Regulates Body Weight and Energy Homeostasis
Source: PLoS Biol. 2013 Mar 12;11(3):e1001506. doi: 10.1371/journal.pbio.1001506 (PMC3595213; doi:10.1371/journal.pbio.1001506)

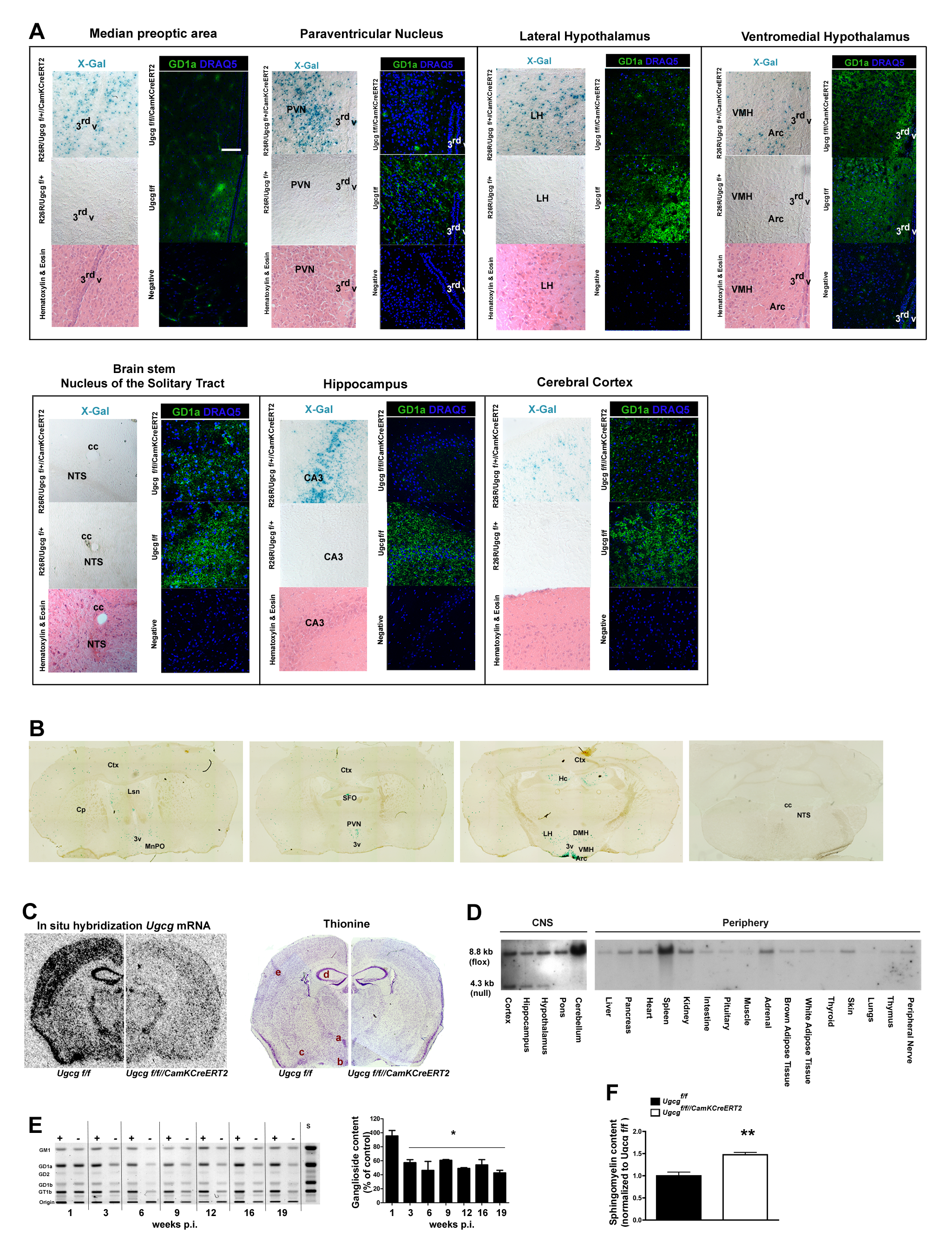

Supplement: Figure S1 — Generation and characterization of inducible Ugcg f/f//CamKCreERT2 mice. (A) R26R/Ugcg f/+//CamKCreERT2 reporter mice and R26R/Ugcg f/+ mice were induced with tamoxifen and brains were removed 3 d after the last injection. X-Gal staining revealed Cre activity in hypothalamic MnPO, paraventricular nucleus (PVN), and lateral hypothalamus (LH). Cre activity was absent in ventromedial hypothalamus (VMH) and in the brain stem nucleus of the solitary tract (NTS; cc, central canal). Cre activity could be detected in hippocampus and cerebral cortex. GD1a immunofluorescence visualized ganglioside depletion in Cre-targeted regions of male Ugcg f/f//CamKCreERT2 mice (6 wk p.i). Scale bar: 100 µm. (B) Overview of brain slices from R26R/Ugcg f/+//CamKCreERT2 reporter mice indicating Cre activity. 3v, 3rd ventricle; CP, caudoputamen; LSN, lateral septal nucleus; SFO, subfornical organ; CTX, cortex; HC, hippocampus; DMH, dorsomedial hypothalamus. (C) In situ hybridization showed depletion of Ugcg mRNA in hypothalamic PVN (a), SCN (b), as well as in the amygdala (c), hippocampus (d), and cerebral cortex (e) of male Ugcg f/f//CamKCreERT2 mice (4 wk p.i.). Respective areas were visualized by thionine staining. (D) Southern blot did not show recombination events in peripheral tissues of female Ugcg f/f//CamKCreERT2 mice 2 wk p.i. The Ugcg null allele could solely be detected in cerebral cortex, hippocampus, and hypothalamus. (E) Stable ganglioside depletion 3 wk p.i. in targeted neuronal populations of totally dissected hippocampus as shown by TLC. Residual gangliosides from nontargeted cells and innervating fibers were still visible. (+ Ugcg f/f; − Ugcg f/f//CamKCre ERT2). Lipid amounts equaling 50 µg of tissue protein were loaded. Quantification from densitometry analysis of thin layer chromatography results is depicted (n = 3). (F) Sphingomyelin was slightly elevated in hippocampus of Ugcg f/f//CamKCreERT2 mice. *p≤0.05; **p≤0.01. Means ± SEM. (TIF) [file pbio.1001506.s001.tif]

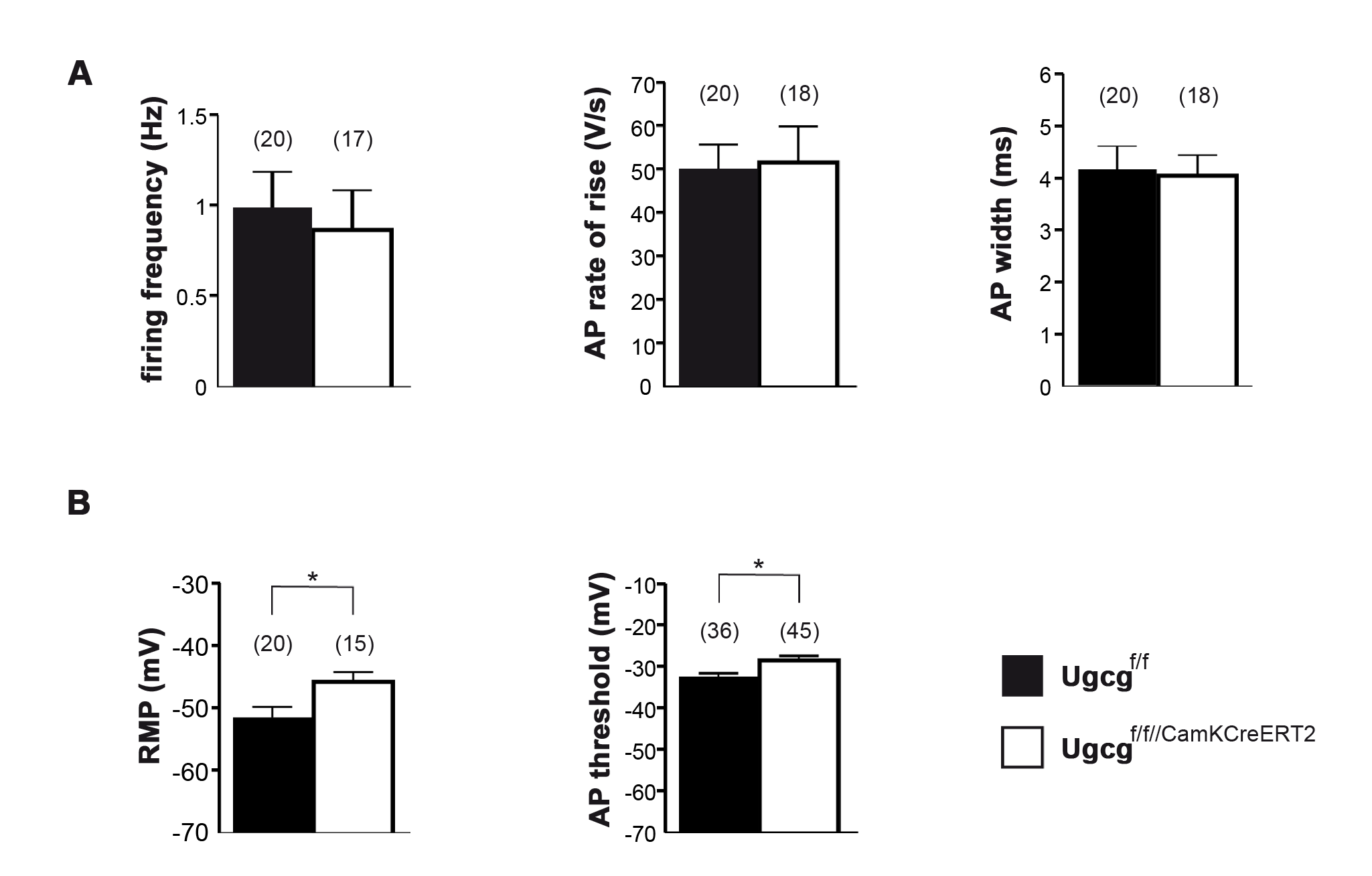

Supplement: Figure S2 — Intrinsic electrical properties of Arc neurons from brain slices of female Ugcg f/f//CamKCreERT2 mice at 12 wk p.i. compared to controls. (A) Spontaneous firing frequency (left), maximal rate of rise of action potentials (AP; middle), and AP width at half-maximal amplitude (right) were not different between both strains. (B) Resting membrane potential (RMP, left) and action potential threshold (right) were both shifted to more depolarized values in cells from Ugcg f/f//CamKCreERT2 mice. Cell numbers are stated in brackets above each column. *p≤0.05. Mean values ± SEM. (TIF) [file pbio.1001506.s002.tif]

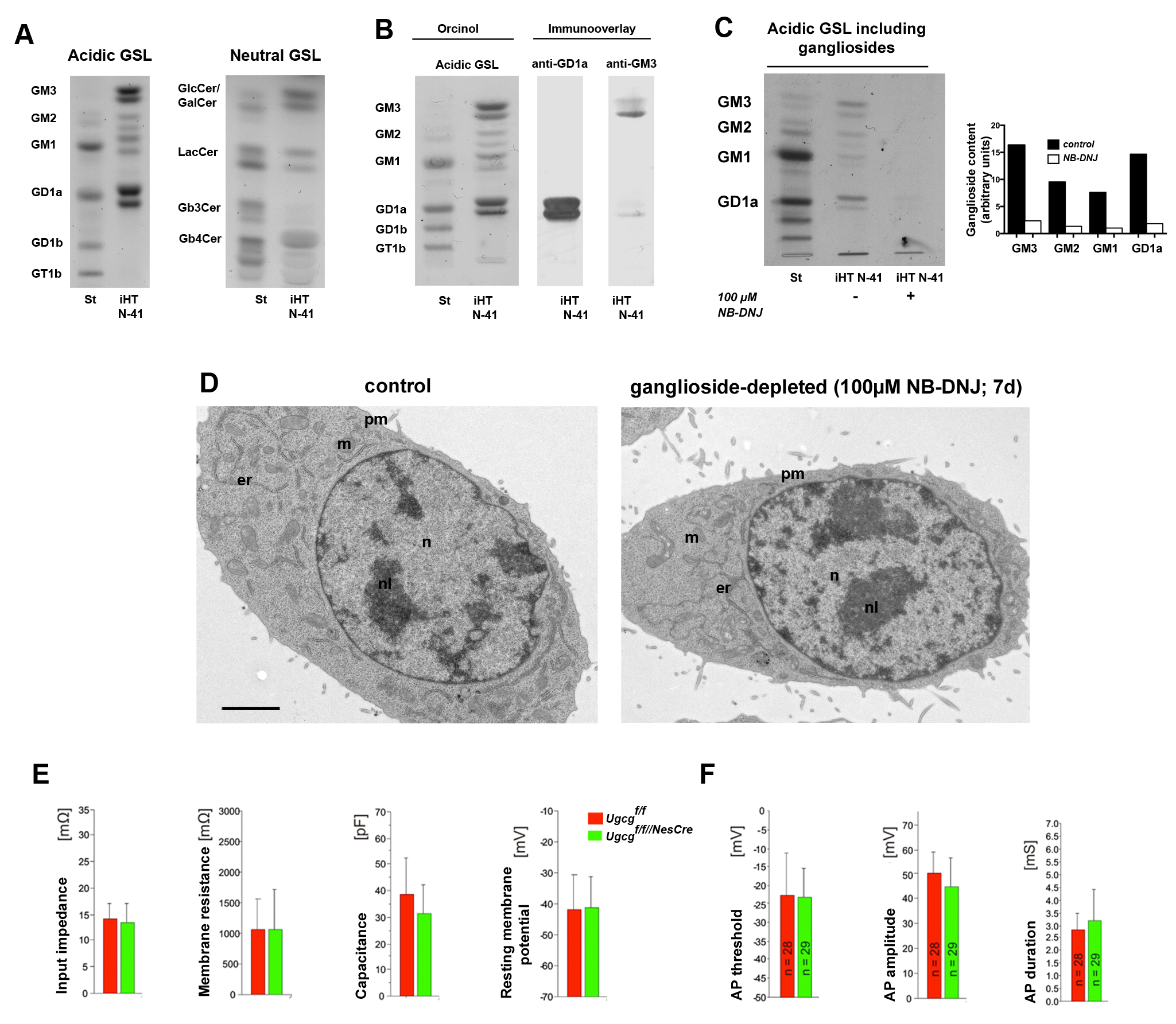

Supplement: Figure S3 — Immortalized hypothalamic cells (N-41 cells) express gangliosides and ganglioside-depleted cells show normal morphological (N-41) and biophysical membrane properties (primary Ugcg f/f//NesCre neurons). (A) Ganglioside expression pattern as determined by TLC after lipid extraction from immortalized hypothalamic cell lines and subsequent separation of neutral and acidic glycosphingolipids including gangliosides (St, standard). (B) Immune overlay TLC confirmed the identity of GD1a and GM3 bands (St, standard). (C) The 7-d treatment of immortalized hypothalamic cells with the GCS inhibitor NB-DNJ (100 µM) led to inhibition of ganglioside biosynthesis, as shown by TLC (St, standard). Quantification of the TLC bands is depicted. (D) Normal membrane appearance and assessed by electronmicroscopy (n, nucleus; nl, nucleoli; m, mitochondria; er, endoplasmic reticulum; pm, plasma membrane). Scale bar: 2 µm. (E). Basic electrophysiological characteristics of primary Ugcg f/f and Ugcg f/f//NesCre neurons from total embryonic hypothalamus (E16–E18). Whole-cell recordings from randomly picked neurons between 7 DIV and 12 DIV. Membrane resistance and capacitance (tested with a 5 mV test pulse) as well as resting membrane potential were unaltered in Ugcg f/f//NesCre neurons. (F) Action potential threshold, amplitude and duration were also unaltered in Ugcg f/f//NesCre neurons. Data are given as mean values ± SEM. (TIF) [file pbio.1001506.s003.tif]

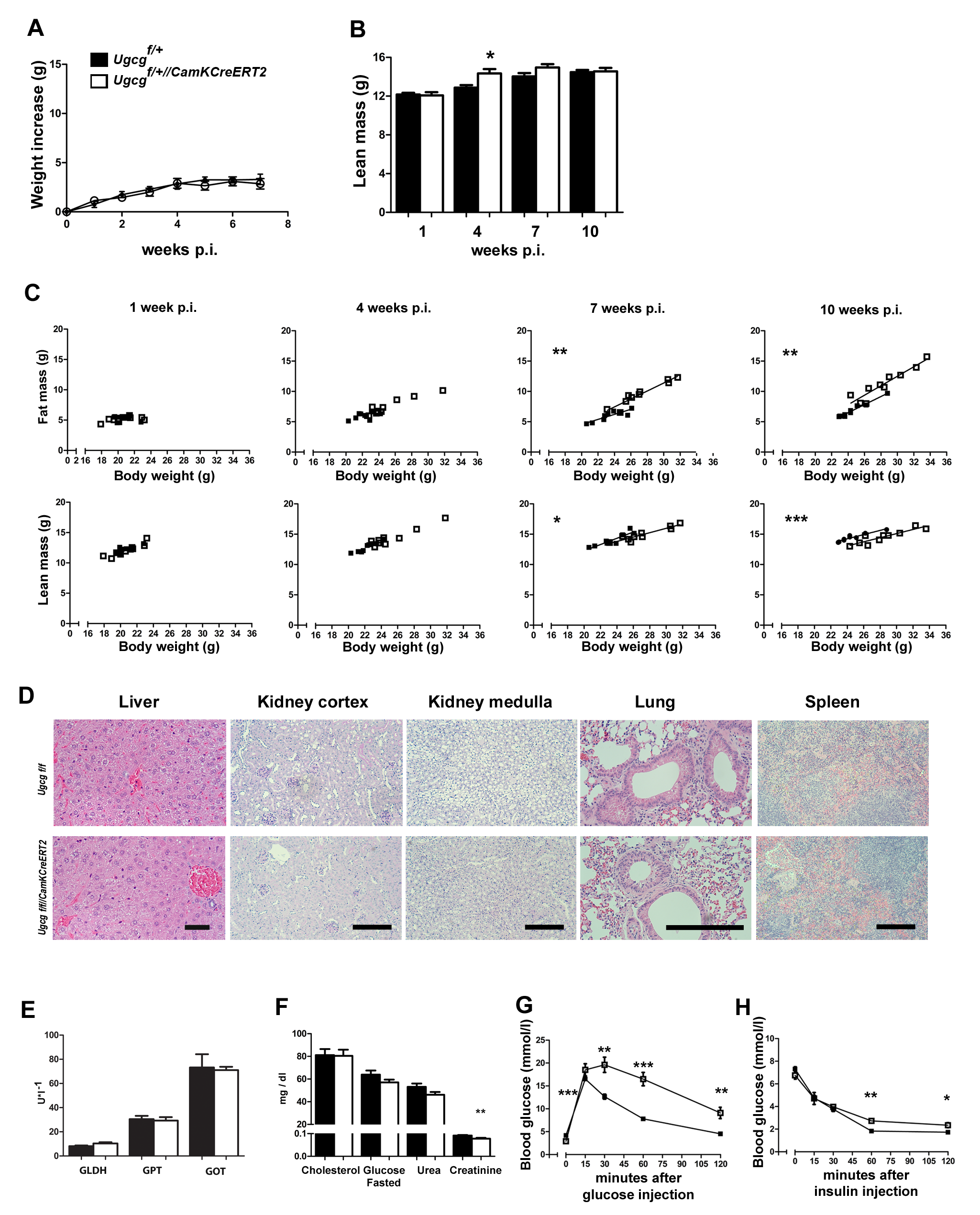

Supplement: Figure S4 — Fat mass in Ugcg f/f//CamKCreERT2 mice was elevated, while organ morphology was unaltered. (A) Unaltered body weight in heterozygous Ugcg f/+//CamKCreERT2 mice (n = 4). (B) NMR analysis revealed that lean mass was only slightly increased 4 wk p.i. (n = 9–10). (C) Significantly increased body fat mass in Ugcg f/f//CamKCreERT2 mice 7 and 10 wk p.i. when adjusted for body weight, as determined by nuclear magnetic resonance imaging (n = 9–10 per group); **p≤0.01 (LM). Individual values. Relative lean mass, when plotted against body weight, decreased during progressive weight gain 7 and 10 wk p.i. in Ugcg f/f//CamKCreERT2 mice (n = 9–10 per group); *p≤0.05; ***p≤0.001 (LM). Individual values. (D) Unaltered morphology of major peripheral organs in female mice was shown by hematoxylin and eosin stainings 9 wk p.i. No liver steatosis could be detected. (Scale bars: Liver, 50 µm; Kidney, Lungs, Spleen, 200 µm). (E) Parameters indicative for liver function did not show any significant changes 19 wk p.i., indicating normal liver function in Ugcg f/f//CamKCreERT2 mice (n = 12; GOT, n = 4). (F) Serum levels for cholesterol, fasted glucose, urea, and creatinine 19 wk p.i. did not show any biologically relevant differences in mice (n = 12). (G) Ugcg f/f//CamKCreERT2 mice displayed impaired glucose tolerance 12 wk p.i. (n = 9; Mann-Whitney Rank Sum Test). (H) Slight but significant insulin insensitivity was detected in Ugcg f/f//CamKCreERT2 mice 10 wk p.i. (n = 8–9; Mann-Whitney Rank Sum Test). *p≤0.05; **p≤0.01; ***p≤0.001. Means ± SEM unless stated otherwise. (TIF) [file pbio.1001506.s004.tif]

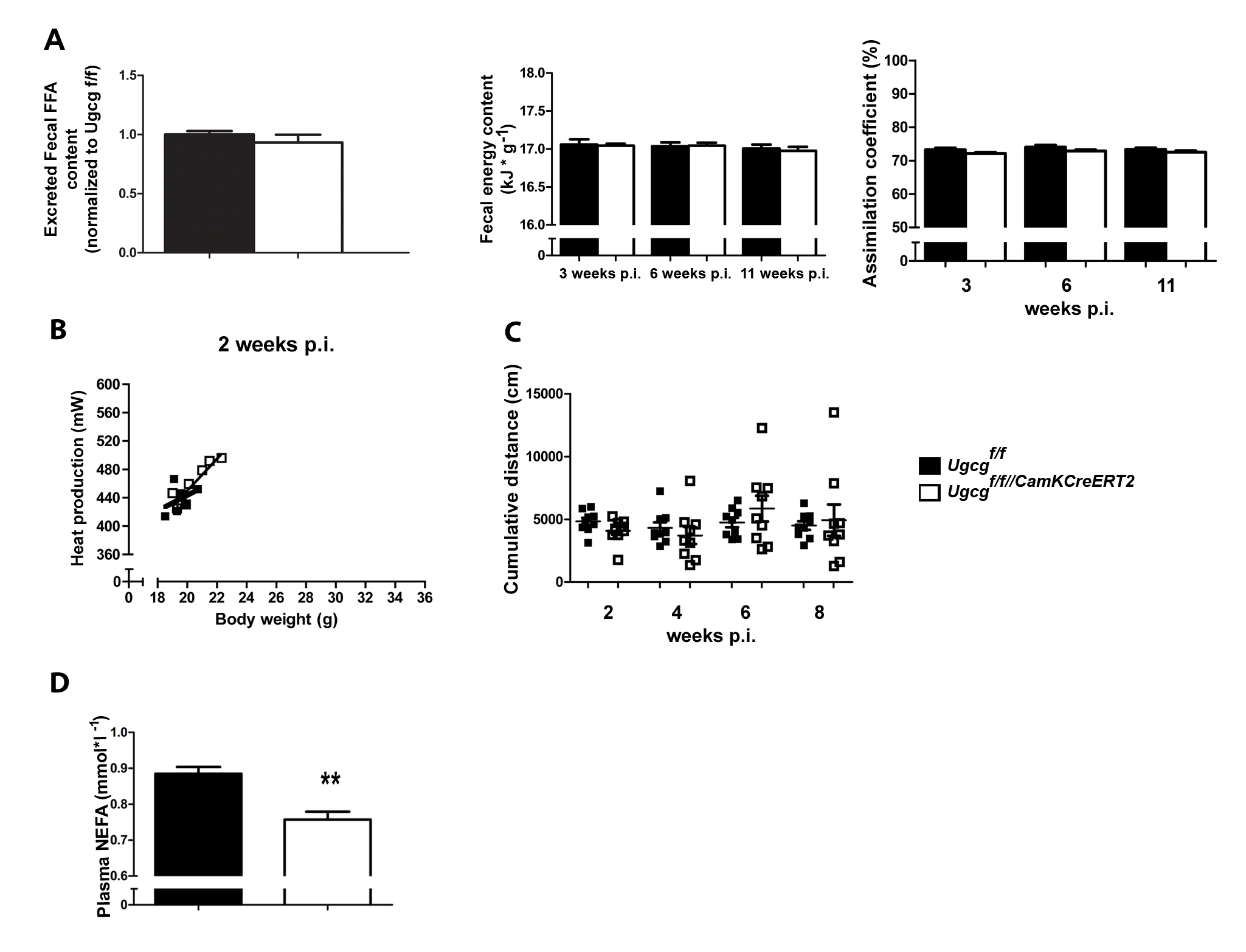

Supplement: Figure S5 — Normal excretion and unaltered spontaneous locomotor activity in Ugcg f/f//CamKCreERT2 mice, but plasma NEFAs are altered. (A) Fecal free fatty acids (8 wk p.i.), fecal energy content, and assimilation coefficient were not altered in Ugcg f/f//CamKCreERT2 mice 3, 6, and 11 wk p.i. Means ± SEM. (B) Metabolic rate of Ugcg f/f//CamKCreERT2 mice was unaltered 2 wk p.i. (n = 7–10). Individual means. (C) Spontaneous locomotor activity measured at different time points after tamoxifen induction did not reveal any statistically significant difference between Ugcg f/f//CamKCreERT2 mice and control littermates (n = 9) (Mann-Whitney Rank Sum Test). Medians and individual values. (D) Decreased plasma NEFA values in fasted Ugcg f/f//CamKCreERT2 mice 11 wk p.i., indicating decreased fat mobilization (n = 4–6). **p≤0.01. Means ± SEM. (TIF) [file pbio.1001506.s005.tif]

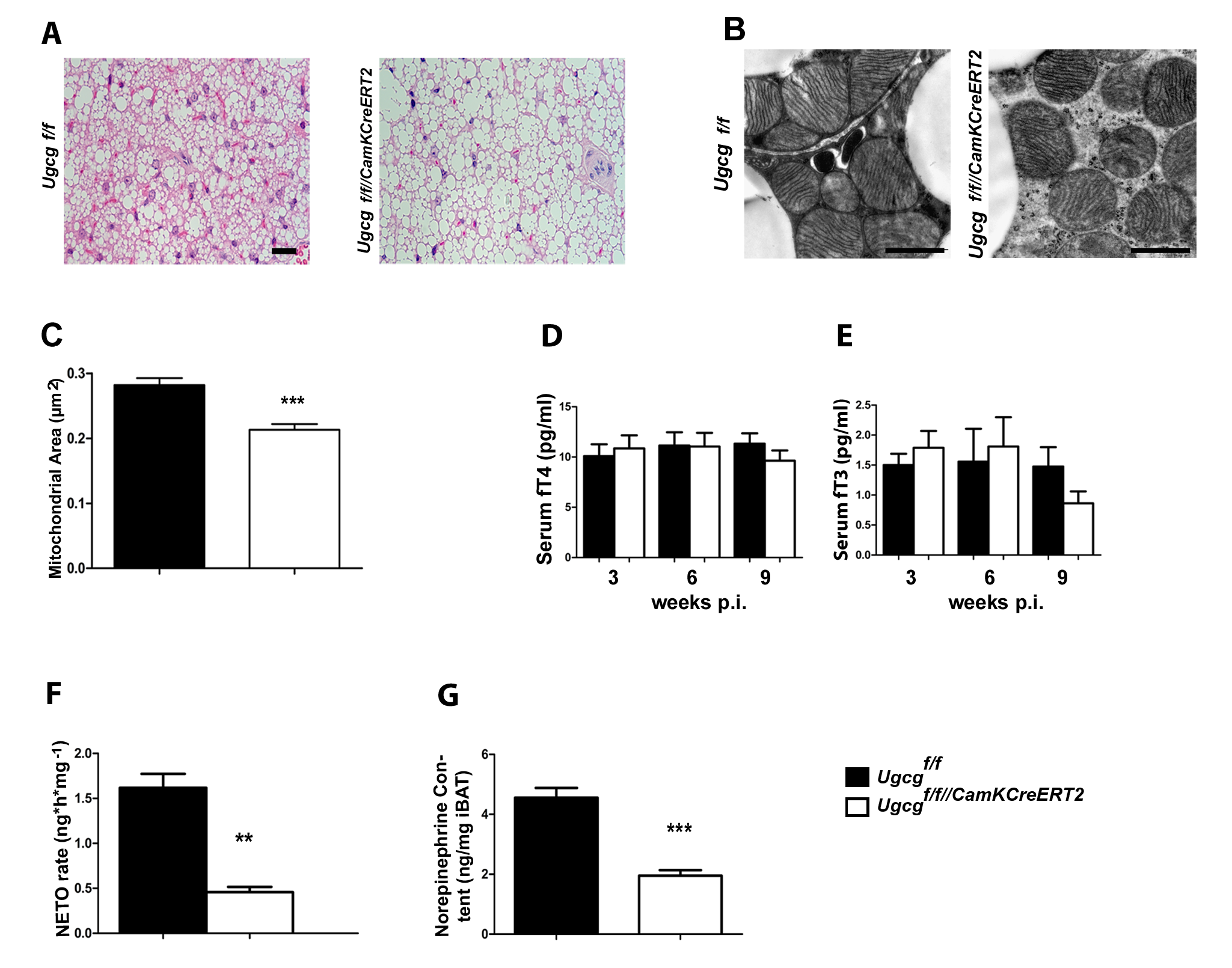

Supplement: Figure S6 — Hypothermia in Ugcg f/f//CamKCreERT2 mice is not due to defective thyroid function, but decreased sympathetic activity in iBAT. (A) Enlarged lipid droplets were found in iBAT of Ugcg f/f//CamKCreERT2 mice 9 wk p.i. (B) Altered mitochondrial density and size were detected in Ugcg f/f//CamKCreERT2 mice by ultrastructural analysis of iBAT 9 wk p.i. (C) Mitochondrial area was reduced in Ugcg f/f//CamKCreERT2 mice (n = 3 mice, 195 mitochondria). (D) Serum-free thyroxine (fT4) was determined by ELISA and did not show any alterations in Ugcg f/f//CamKCreERT2 mice (n = 5–6 per group). (E) Serum-free triiodothyronine (fT3) was determined by ELISA and did not show any alterations in Ugcg f/f//CamKCreERT2 mice (n = 3–6 per group). (F) High performance liquid chromatography (HPLC) revealed lower sympathetic activity (NETO rate) per mg iBAT 9 wk p.i. (n = 4). (G) NE content was also decreased in Ugcg f/f//CamKCreERT2 iBAT 9 wk p.i. (n = 4). **p≤0.01; ***p≤0.001. Means ± SEM. (TIF) [file pbio.1001506.s006.tif]

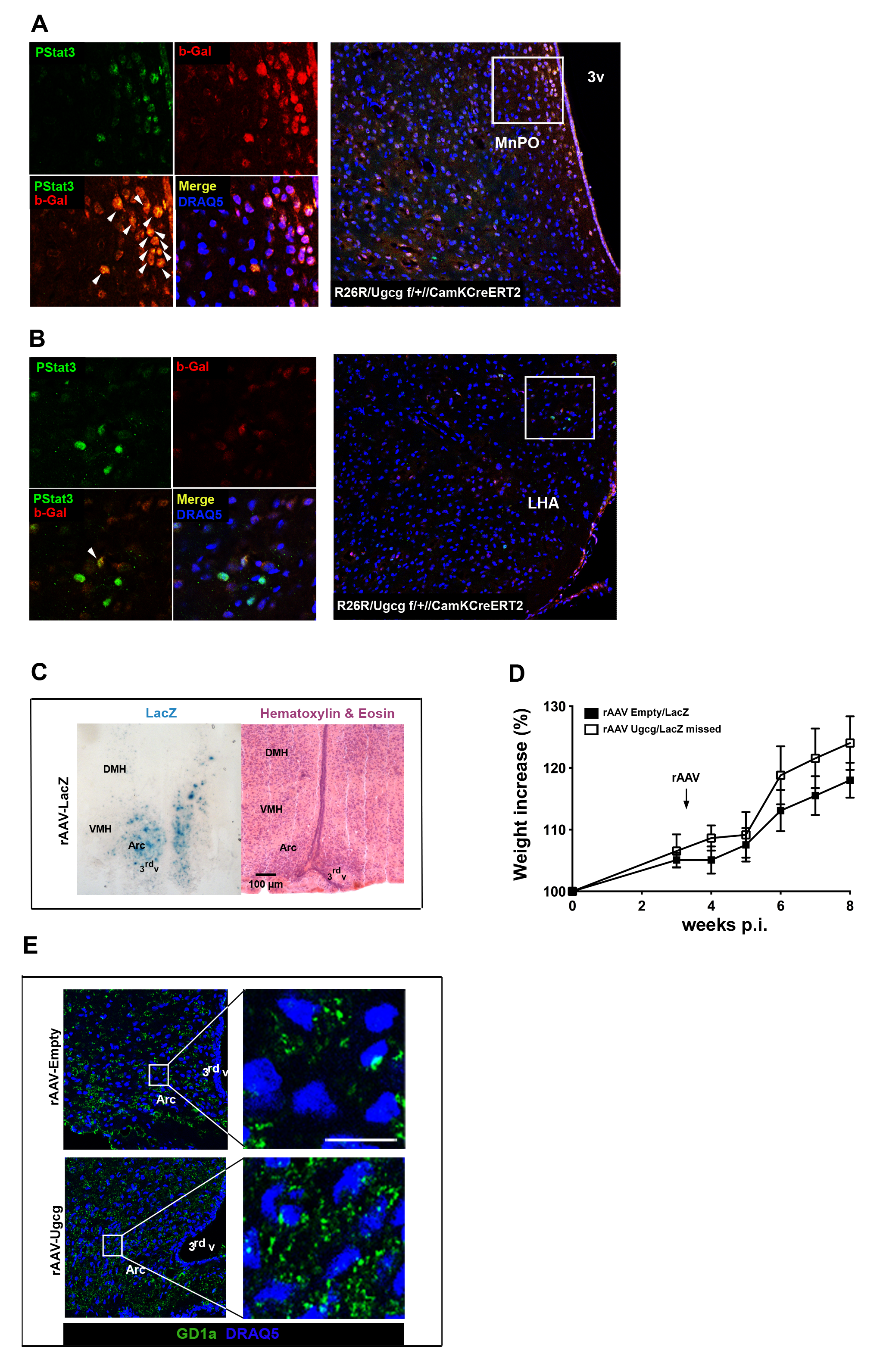

Supplement: Figure S7 — rAAV-mediated Ugcg gene delivery to the hypothalamic Arc ameliorates obesity in Ugcg f/f//CamKCreERT2 mice. (A and B) Double immunofluorescence showed that Cre activity, indicated by beta galactosidase staining (b-gal), was targeted to MnPO neurons (A) expressing the long form of the ObR (see arrowheads), but not to the majority of ObR neurons in the LHA (B), as indicated by PStat3 staining in leptin-injected R26R/Ugcg f/+//CamKCreERT2 mice (5 mg/kg leptin, 120 min). (C) Stereotactic rAAV-LacZ delivery to the Arc was exemplarily demonstrated by X-Gal-staining. Morphology was depicted in the HE section. (D) Ugcg f/f//CamKCreERT2 mice that were not targeted in the Arc by stereotactic delivery of rAA viruses encoding Ugcg and lacZ did not show improvement in body weight increase compared to rAAV-Empty/lacZ-injected Ugcg f/f//CamKCreERT2 mice (n = 4 rAAV-Ugcg/lacZ missed, n = 8 rAAV-Empty/lacZ). The graph depicting rAAV-Empty/lacZ-targeted mice is taken from Figure 4B for comparison. (E) Restored ganglioside biosynthesis in the Arc of rAAV-Ugcg-injected Ugcg f/f//CamKCreERT2 mice, as shown by GD1a immunofluorescence 8 wk p.i. Shown are overview pictures for Figure 4G (also taken for Figure S7E). Scale bar: 18 µm. Means ± SEM. (TIF) [file pbio.1001506.s007.tif]

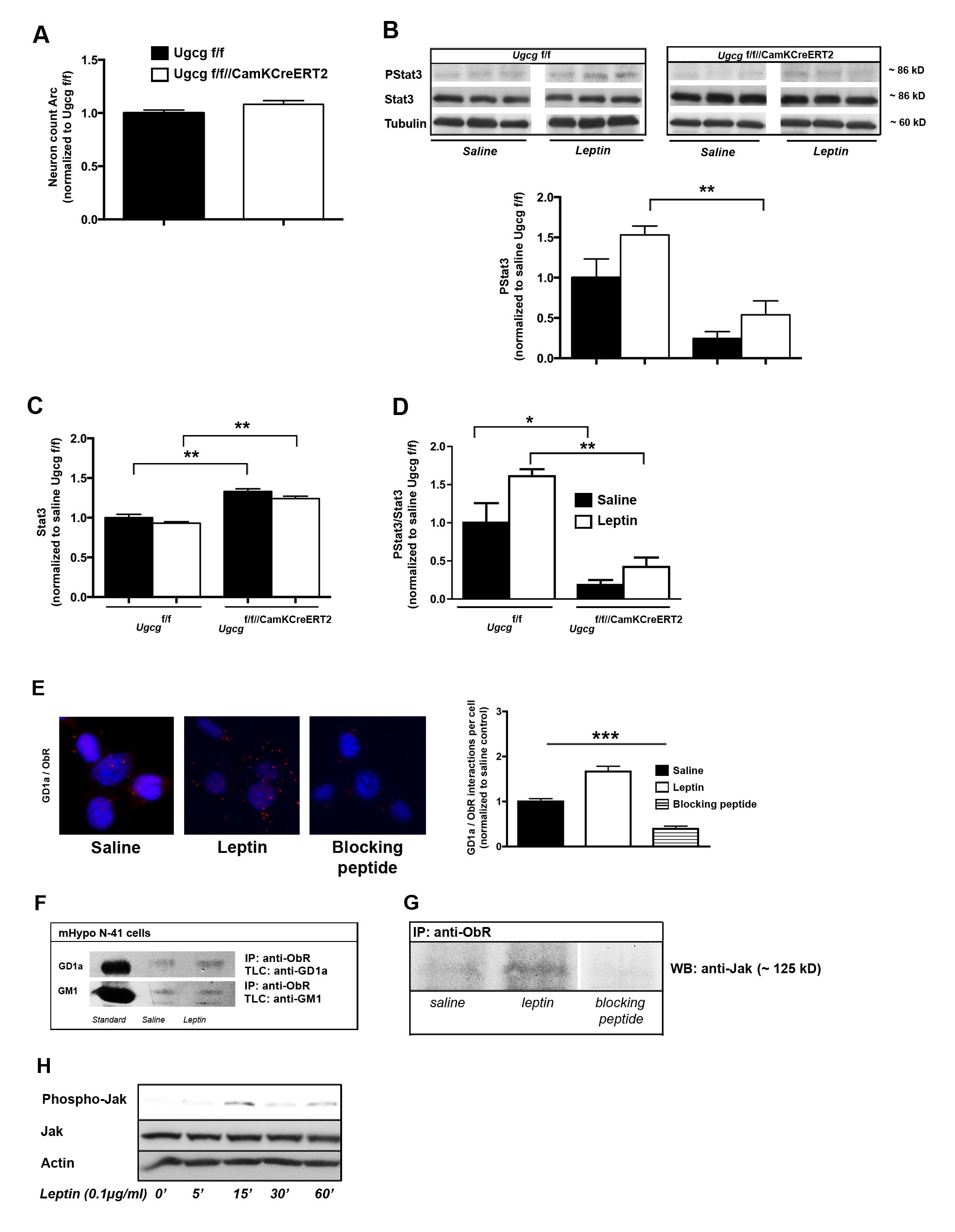

Supplement: Figure S8 — GCS in hypothalamic neurons regulates neuronal leptin signaling. (A) Neuron count in the Arc was normal in Ugcg f/f//CamKCreERT2 mice (n = 115–122 sections). Quantification contains normalized data from 12 mice each. (B) Stat3 phosphorylation in MBH of male mice was investigated by Western blot 6 wk p.i. We loaded 100 µg of protein in each lane. Elevated PStat3 levels were not seen in Ugcg f/f//CamKCreERT2 mice upon leptin stimulation. Quantification for PStat3/Tubulin is depicted (n = 3). (C) Baseline and leptin-stimulated Stat3 levels were elevated in Ugcg f/f//CamKCreERT2 mice. Stat3 was normalized for tubulin expression (n = 3). (D) The PStat3/Stat3 ratio is decreased in Ugcg f/f//CamKCreERT2 mice 6 wk p.i. both at baseline and after leptin stimulation. PStat3 and Stat3 levels were normalized for tubulin (n = 3). (E) Immortalized hypothalamic cells (N-41 cells) were incubated with either saline or 100 ng/ml leptin (10 min). A proximity ligation assay (PLA; principle depicted in Figure 5E) for GD1a/ObR similar to the experiment depicted in Figure 5F was performed using a different ObR-specific antibody. GD1a/ObR interactions were detected and leptin treatment dynamically increased the GD1a/ObR PLA spots per cell similar to the result depicted in Figure 5F. Pre-adsorption of the antibody by a blocking peptide abolished PLA signals (n = 74–150 cells). (F) Similar to the experiments depicted in Figure 5H, extracts from saline- and leptin-treated N-41 cells were immunoprecipitated with a second ObR antibody, lipids were extracted, and GD1a and GM1 were visualized by immune overlay TLC. Similar to the results obtained in Figure 5H, GD1a and GM1 co-immunoprecipitated (Co-IP) with ObR. (G) Similar to the result in Figure 5I, Jak was co-precipitated with the ObR, which increased upon leptin stimulation of N-41 cells, using a second ObR antibody. Addition of the blocking peptide abolished the signal. (H) Weak Jak phosphorylation was induced in N-41 cells after 30 min of l [file pbio.1001506.s008.tif]

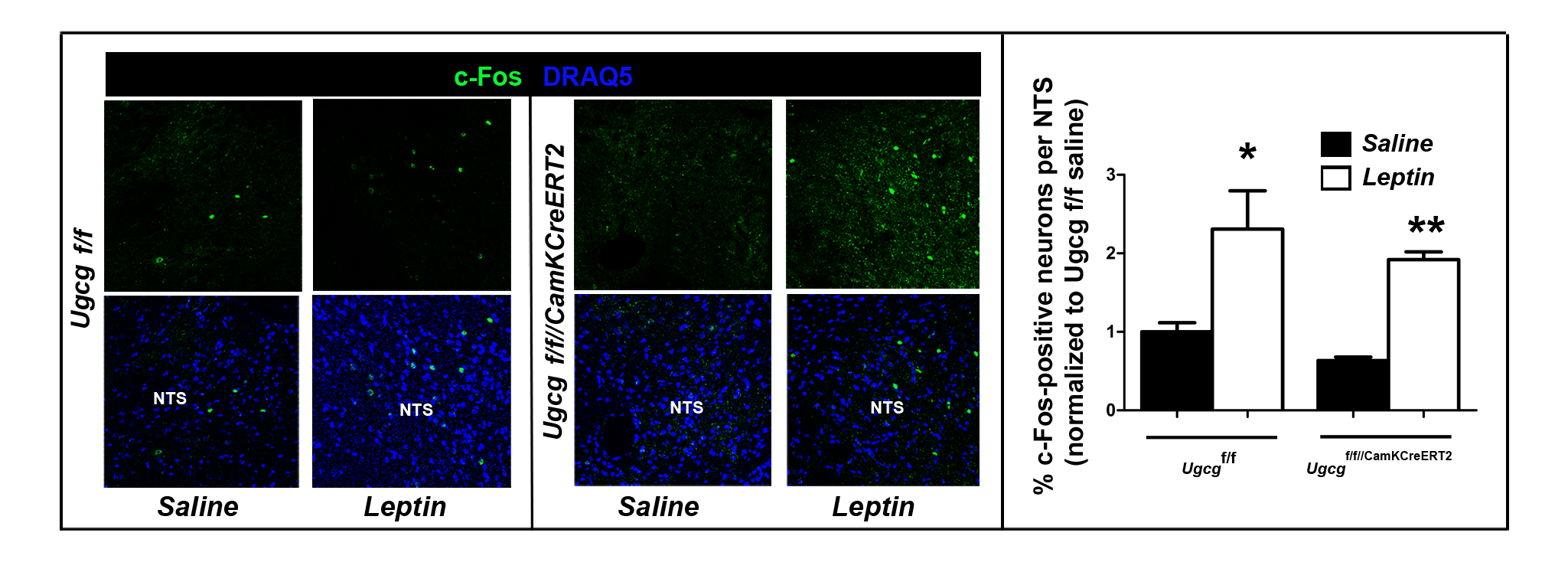

Supplement: Figure S9 — The brain stem NTS of Ugcg f/f//CamKCreERT2 mice retained leptin responsiveness 6 wk p.i. c-Fos expression was unaltered in the nontargeted NTS (n = 2–4 sections). Datasets for each time point were acquired individually. Immunofluorescence and image acquisition for each dataset (treated and untreated controls and knockouts) were performed simultaneously. *p≤0.05; **p≤0.01. Means ± SEM. (TIF) [file pbio.1001506.s009.tif]

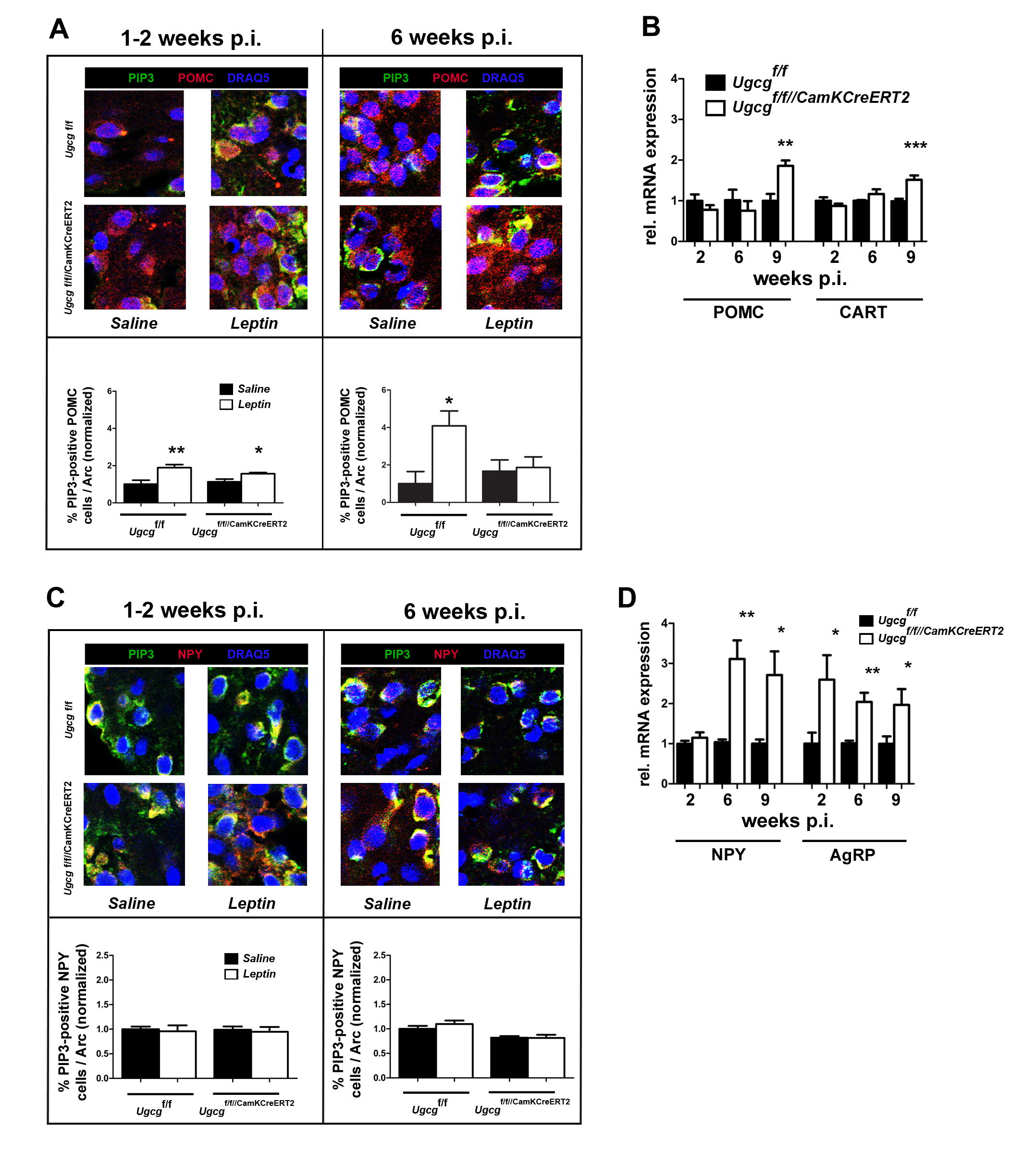

Supplement: Figure S10 — Reduced PIP3 formation in POMC neurons of Ugcg f/f//CamKCreERT2 mice upon leptin stimulation. (A) Fasted mice were injected with either saline or leptin (5 mg/kg body weight) and sacrificed 45 min later. Either POMC or NPY staining identified individual neuronal populations. Leptin evokes PIP3 formation in Arc POMC neurons of Ugcg f/f mice as well as Ugcg f/f//CamKCreERT2 mice before ganglioside depletion was completed (1–2 wk p.i.). This response was blunted in ganglioside-depleted POMC neurons 6 wk p.i. The percentage of POMC/PIP3-double-positive neurons per Arc section normalized to Ugcgf/f saline is depicted (n = 3–7). (B) Unaltered mRNA expression of the anorexigenic neuropeptides POMC and CART in the MBH of Ugcg f/f//CamKCreERT2 mice 6 and 9 wk p.i. (n = 3–6). (C) Fasted mice were injected with either saline or leptin (5 mg/kg body weight) and sacrificed 45 min later. NPY staining identified individual neuronal populations. Leptin did not directly lead to increased PIP3 formation in NPY neurons in neither of the groups (n = 3–7). (D) Increased Npy and Agrp mRNA expression in the MBH of Ugcg f/f//CamKCreERT2 mice 6 and 9 wk p.i. (n = 3–6). Datasets for each time point were acquired individually. Immunofluorescence and image acquisition for each dataset (treated and untreated controls and knockouts) were performed simultaneously. n = 3–7 sections; *p≤0.05; **p≤0.01; ***p≤0.001. Means ± SEM. (TIF) [file pbio.1001506.s010.tif]
