# Supplementary material for: Neuronal Expression of Glucosylceramide Synthase in Central Nervous System Regulates Body Weight and Energy Homeostasis
Source: PLoS Biol. 2013 Mar 12;11(3):e1001506. doi: 10.1371/journal.pbio.1001506 (PMC3595213; doi:10.1371/journal.pbio.1001506)
Supplement: Text S1 — Supplemental experimental procedures and supplemental references. (DOC) [file pbio.1001506.s011.doc]

**Supporting Information**

**Neuronal expression of glucosylceramide synthase in central nervous system regulates body weight and energy homeostasis**

*Viola Nordström^1,*^, Monja Willershäuser^2,8^, Silke Herzer^1^, Jan Rozman^2,3,8^, Oliver von Bohlen und Halbach^4^, Sascha Meldner^1^, Ulrike Rothermel^1^, Sylvia Kaden^1^, Fabian C. Roth^6^, Clemens Waldeck^6^, Norbert Gretz^5^, Martin Hrabě de Angelis^2,7,8^, Andreas Draguhn^6^, Martin Klingenspor^3^, Hermann-Josef Gröne^1,9^, and Richard Jennemann^1,9^*

^1^ Department of Cellular and Molecular Pathology, German Cancer Research Center, Heidelberg, Germany

^2^ German Mouse Clinic, Institute of Experimental Genetics, Helmholtz Zentrum München, Neuherberg, Germany

^3^ Molecular Nutritional Medicine, Else-Kröner Fresenius Center, Technische Universität München, Freising-Weihenstephan, Germany

^4^ Institute for Anatomy and Cell Biology, University of Greifswald, Greifswald, Germany

^5^ Medical Research Center, Heidelberg University, Heidelberg, Germany

^6^ Institute for Physiology and Pathophysiology, Heidelberg University, Heidelberg, Germany

^7^ Chair of Experimental Genetics, Technische Universität München, Freising-Weihenstephan, Germany

^8^ Member of German Center for Diabetes Research (DZD), Neuherberg, Germany

^9^ Equally shared last authorship

** Correspondance:*

*v.nordstroem@dkfz-heidelberg.de (tel. +49 6221 42 4356, fax +49 6221 42 4352)*

**Supplemental Experimental Procedures**

**Animal Care**

Mice were housed in groups of up to five animals at 22°C at a 12 hr light / 12 hr dark cycle. For metabolic characterizations, mice were housed individually on wood shavings as litter with a paper tissue provided as bedding material and they were kept at room temperature (23±1°C) at a 12 hr light / 12 hr dark cycle (lights on 6:00 am CET). Animals were fed regular lab chow diet (Kliba Nafag, Switzerland). Animals were given *ad libitum* access to water and food all times. Food withdrawal was indicated if required for an experiment.

**Genotyping of *Ugcg*^f/f//CamKCreERT2^ and R26R Mice**

Mice were genotyped by PCR using DNA acquired from tail tips. The following primers were used: 5’-GATCTAAGAGGGTGAAGGCGCA-3’ (*Ugcg* wild type forward); 5’-AAGCCAGTCCAGTCAAACCGAG-3’ (*Ugcg* wild type/ flox reversed);5’-CTGCCTTGCAATCCTGTCTGTC-3’ (*Ugcg* null reversed); 5’-GGTTCTCCG-TTTGCACTCAGGA-3’ (*CamKCreERT2* primer 1); 5’-CTGCATGCACGGGAC-AGCTCT-3’ (*CamKCreERT2* primer 2); 5’-GCTTGCAGGTACAGGAGGTAGT-3’ (*CamKCreERT2* primer 3); 5’-TCTGCTGCCTCCTGGCTTCTGA-3’ (R26R primer 1); 5’-CCAGATGACTACCTATCCTCCCA-3’ (R26R primer 2); 5’-AAGCGCATGC-TCCAGACTGCCT-3’ (R26R primer 3).

**Indirect Calorimetry and Determination of Metabolizable Energy**

Metabolic measurements (oxygen consumption and carbon dioxide production) were performed using an open circuit respirometry system (SM-MARS; Sables Systems, USA) that allowed the serial measurement of up to 7 mice and one empty control channel. The air was pulled through the metabolic cages with a flow rate of 54l/h. Basics of gas analysis and open-circuit calorimetry are described in detail elsewhere.

For the gas analysis mice were individually kept in metabolic cages (~4.1l) with paper tissue provided as bedding material and food and water *ad libitum*. Metabolic cages were set up in a ventilated cabinet continuously supplied with an overflow of fresh compressed air. Oxygen consumption (VO_2_) and carbon dioxide (CO_2_) production of each mouse were analysed in 10 min intervals and monitored for 21 h (total resolution of 126 data points per mouse) beginning in the early afternoon (1:00 pm CET) until the next morning (10:00 am CET). The simultaneous analysis of oxygen consumption and carbon dioxide production allowed the calculation of the respiratory exchange ratio (RER = VCO_2_/VO_2_) and heat production (HP) using the following equation: (4.44 + 1.43*RER)*VO_2_ (Heldmaier and Steinlechner, 1981). For body weight dependent analysis the individual mean values of the whole 21 h measurement were taken. To control for steady state conditions and to account for differences the body weight of the mice was recorded prior and after the measurement.

For the analysis of food intake and metabolizable energy mice were individually housed on grid panels (0.5°cm grid hole diameter). An igloo (Mouse Igloo, red, BIO-SERV) was provided as shelter. Mice were acclimatized to the conditions for three days prior to start of the experiment. In the following experiment period of 5-9 days body weight and food intake were determined daily. After the trial feces and spillage of food in the cages were collected, dried in an oven at 60°C for one day and then separated from each other. Food intake was corrected for spillage and the feces were dried for another two days at 60°C until there was no further change in mass. After the drying procedure feces and lab chow (also dried for 2 days at 60°C) were homogenized in a coffee grinder. Approximately 1g of each sample was squeezed to a pill for determination of energy content in a bomb calorimeter (IKA-Calorimeter C7000, IKA- Analysentechnik, Germany). EMET was determined as follows: Energy uptake was calculated as the product of food intake and the caloric value of the food determined by bomb calorimetry (IKA-calorimeter C7000, IKA-Analysetechnik), and the energy excreted via feces and urine (approximated 2%;) was subtracted from total energy uptake. The assimilation coefficient was calculated as follows: (E_MET_*100)/energy intake.

Whole body composition was determined by non-invasive NMR analysis (Mini-Spec, Bruker Optics).

Total spontaneous locomotor activity in a novel environment was assessed in an ActiMot system (TSE Systems, USA) by 60 min measurements using 265x205x140 mm cages.

**Glucose tolerance and insulin sensitivity test**

The intraperitoneal (i.p.) glucose tolerance test was performed following an overnight fast of approximately 15 h (food removed at ~ 5:00 pm CET). Fasting blood glucose levels were analysed prior to the intraperitoneal injection of 2 g/kg glucose (Glucose 20%, Braun, Germany). Blood glucose levels were determined 15, 30, 60 and 120 min after glucose administration from a drop of blood from the tail vein using a hand hold glucometer (Accu Check, Aviva, Roche).

For insulin sensitivity measurements mice were food restricted 4 h prior to the experiments (food removed at ~9:30 CET). Baseline glucose levels were determined before mice were injected i.p. with 0.75 units/kg human insulin (Lilly, Germany). Glucose levels were determined 15, 30, 60, and 90 min after insulin injection as described above.

**Determination of Norepinephrine Turnover Rate (NETO) and Norepinephrine Content in iBAT**

NETO rate in iBAT was determined by the α-methyl-p-tyrosine (AMPT) method as described earlier . In brief, animals were divided into two groups: untreated (n = 4 / 4; *Ugcg*^f/f^ / *Ugcg*^f/f//CamKCreERT2^) and AMPT-treated (n = 4 / 4; *Ugcg*^f/f^ / *Ugcg*^f/f//CamKCreERT2^). Untreated animals were killed by cervical dislocation at 0h to obtain baseline NE values. The second group of animals was injected with 250 mg/kg of a 25 mg/ml AMPT solution. A supplemental dose of AMPT (125 mg/kg at a concentration of 12.5 mg/ml) was administered to the animals at 2 h after the initial injection to maintain inhibition of catecholamine biosynthesis. The animals were sacrificed at 4 h after the initial AMPT injection and iBAT was rapidly removed, weighed and shock-frozen in liquid nitrogen.

The norepinephrine tissue content was measured using reversed-phase high performance liquid chromatography with electrochemical detection (Chrome Systems, Germany) as described earlier (Brito et al., 2007). Briefly, tissues were thawed and homogenized in 0.2 M perchloric acid containing 1 mg/ml ascorbic acid and dihydroxybenzylamine (DHBA; 100 ng per 50 mg iBAT) as internal standard. After centrifugation of samples (7500 x g, 15 min at 4°C), catecholamines were extracted from the supernatant with alumina and eluted into the perchloric acid / ascorbic acid. NETO rate was calculated as described earlier (Brito et al, 2007). Calculations were made using the following formula: k = (lg[NE]_0_ – lg[NE]_4_/(0.434*4) and K = k[NE]_0_, where k is the constant rate of norepinephrine (NE) efflux, [NE]_0_ is the initial norepinephrine concentration, [NE]_4_ is the final NE concentration, and K = NETO. The slopes of all possible combinations of animals were calculated and used to determine the NETO rate.

**Organ Morphology**

Organs destined for morphological analysis were dissected and fixed in 4% paraformaldehyde for paraffin preparation. Paraffin-embedded tissues were used for hematoxylin & eosin stainings as well as periodic acid-Schiff stainings (PAS).

**Tissue Dissection, RNA Isolation and Quantitative mRNA Expression Analysis**

In order to analyze neurotransmitter expression, non-fasted mice were sacrificed and mediobasal hypothalami were dissected between 3 p.m. – 5 p.m.to reduce variations in circadian expression levels. Total RNA was extracted from hypothalami as described earlier . In brief, frozen samples were homogenized in GTC buffer (4.23M guanidine-thiocyanate, 25mM citrate, 0.2M β-mercaptoethanol) and RNA was isolated by addition of 0.1 vol 2M sodium acetate, 1 vol phenol, 0.2 vol (24:1 chloroform:isoamylalcohol) and precipitated with isopropanol at -20°C.

Arcuate nuclei for SOCS-3 expression analysis were microdissected from non-fasted mice with the help of a stereomicroscope. Mamillary nuclei as well as the third ventricle and optical chiasm were landmarks for standardized excision of arcuate nucleus-enriched hypothalamic tissue. RNA was isolated using TRIzol® (Invitrogen, USA) according to the manufacturer’s instructions. Total nucleic acid content was measured spectrophotometrically and reversely transcribed using Superscript II Reverse Transcriptase (Invitrogen, Germany) according to the manufacturer’s guidelines. Quantitative real-time PCR was carried out with help of the LC FastStart DNA Master SYBR Green I kit (Roche) and the Light Cycler® (Roche) and the following primers:

*Tubulin* 5’-TCTCTCACCCTCGCCTTCTA-3’ (forward), *Tubulin* 5’- GGGTTCCAG-GTCTACGAACA-3’(reversed); *Agrp* 5’-GTCTAAGTCTGAATGGCCTCAAG-3’ (forward), 5’-CATCCATTGGCTAGGTGCGAC-3’(reversed); *Pomc* 5’-CACGTG-GAAGATGCC-GAGAT-3’(forward), 5’-TCCAGCGAGAGGTCGAGTTT-3’(reversed); *Npy* 5’-AGATACTACTC-CGCTCTGCGACA-3’(forward), 5’-TTTCAGGGGATGAGA-TGAGATGA-3’(reversed); *Cart* 5’-ACGAGAAGAAGTACGGCCAAGTC-3’ (forward), 5’-CCTTCACAAGCACTTCAAGAGGA-3’(reversed); 5’-CCCGATAATCTCCATCAG-TTTCC-3’(reversed); *Leprb* (“long form”) 5’-AGAATGACG-CAGGGCTGTATGT-3’(forward), 5’-TCACTGATTCTGCAT-GCTTGGT-3’(reversed); *Socs-3* 5’-GGGTTTTTATGC-TGGCCAAA-3’(forward), 5’-AAGGGATCTGCGAGG-TTTCA-3’(reversed). Expression levels were normalized for tubulin expression.

**Generation of rAAV**

The AAV Helper-free System (Agilent Technologies Inc.) was used for preparation of viruses. Full-length mouse *Ugcg* cDNA was cloned into the pAAV-MCS vector from the Helper-free System (pAAV-Ugcg). Primers for cloning including a His-Tag at the C-terminus of the enzyme: 5’-AGCTTAGGCGTACAATCCGTATCTAGAATGGCG-CTGCTGGACCTG-3’ (forward), 5’ CCCAAGCTTTTAATGGTGATGGTGATGGTGC-ACATCCAGGATCTCCT-3’ (reversed). pAAV-Ugcg, pHelper and pAAV-RC plasmids were co-transfected into AAV-239 cells to generate rAA viruses harvesting *Ugcg* (rAAV-Ugcg) according to the manufacturer’s guidelines. 72 h post transfection, cells were harvested and rAAV-Ugcg from disintegrated cells were immediately purified by ultracentrifugation in an iodixanol step gradient (15%, 25%, 40%, 60% iodixanol) as previously described . Viruses were then concentrated (Amicon concentrator, Millipore), dissolved in PBS and stored at -80°C until injection. Viral titers were approximated by quantification of Ugcg copies using the LC FastStart DNA Master SYBR Green I kit (Roche) according to the manufacturer’s guidelines and the Light Cycler® (Roche). In parallel, rAAV containing the empty pAAV-MCS (rAAV-Empty) and rAAV containing pAAV-LacZ (rAAV-LacZ) were prepared.

***In situ* hybridization and southern blot**

Mice were sacrificed, total brains were dissected and frozen on dry ice (n = 2). 14µm cryostat sections were prepared. Each slide was subsequently incubated with 0.006 pmol of oligonucleotide (5’-GATCTAGCACATCCTTCCTCATCAAACAAG-ACATCCCCGTCACAC-3’) labelled with [α^33^P]dATP by the terminal deoxynucleotidyl transferase (TdT) method according to the manufacturer’s instructions (Boehringer Ingelheim, Germany). Hybridization occurred over night at 42°C. After subsequent washing steps in SSC buffer (0.15M NaCl, 0.015M sodium citrate), 70% ethanol, and 90% ethanol, slides were air-dried and probe hybridization was visualized by exposure to an X-ray film (AGFA, Germany). After film development, slides were counterstained with 0.1% thionine to reveal morphological details. Southern blot analysis of isolated DNA from investigated tissues was carried out according to standard procedures as described earlier .

**Blood Parameters and serum hormone levels**

Serum was prepared from fasted animals between 8.00 a.m. – 10.00 a-m. Whole blood was withdrawn from the retroorbital bulbus and allowed to clot for 30 min at room temperature. Erythrocytes were sedimented by centrifugation and the serum supernatant was collected and stored in aliquots at -80°C.

Serum creatinine (enzymatic determination), urea cholesterol, and liver enyzmes were determined by a Hitachi 9-17-E autoanalyzer (Hitachi, Frankfurt/M, Germany) as described earlier .

Serum hormone levels were determined using commercially available ELISA kits according to the manufacturer’s guidelines (human-fT3-ELISA, human-fT4-ELISA, (Alpha Diagnostic)).

**Transmission Electronmicroscopy**

Tissues were dissected and immediately fixed in Karnovsky solution in 0.2M cacodylate buffer, pH 7.4 and embedded in Araldite (Serva). Ultrathin sections (70 nm) were stained with lead citrate and uranyl acetate. Photographs were taken with a digital camera mounted on an electron microscope (Zeiss EM 910, Zeiss). Morphometry was done with ImageJ software.

**Primary neuronal cell culture**

*Ugcg*^f/f//NesCre^ mice were generated and genotyped as described . Embryos were taken from pregnant mice at E16-E18. Hippocampi were dissected and the tissue was homogenized with a pipette tip. Approximately 150.000 cells were seeded onto poly-L-lysine-coated coverslips and incubated in Neurobasal® medium (Invitrogen) containing B-27 supplement (Invitrogen), 0.5 mM L-glutamine, and 12.5 µM glutamate. After 3 days a part of the medium was replaced with medium devoid of glutamate. Electrophysiology was carried out 9 DIV.

**Electrophysiology of brain slices from *Ugcg*^f/f//CamKCreERT2^ mice**

Mice were deeply anesthetized using isoflurane (Baxter, Unterschleissheim, Germany) and quickly decapitated. Brains were removed into ice-cold cutting solution, containing (in mM): NaCl 87, KCl 2.5, NaH_2_PO_4_ 1.25, MgCl_2_ 7, CaCl_2_ 0.5, glucose 25, sucrose 75, NaHCO_3_ 26, bubbled with 95% O_2_/5% CO_2_. Coronal sections of 250 µm thickness containing the arcuate nucleus were cut using a vibratome (Leica VT1000S, Nussloch, Germany). Slices were transferred into extracellular solution (32°C) containing (in mM): NaCl 124, KCl 3, NaH_2_PO_4_ 1.25, CaCl_2_ 1.6, MgSO_4_ 1.8, glucose 10, NaHCO_3_ 26, bubbled with 95% O_2_/5% CO_2_. After at least 1 h of recovery, slices were transferred into a recording chamber under an upright microscope (Olympus BX51WI, Hamburg, Germany) perfused with extracellular solution at 32°C. Arcuate neurons were identified at 40x magnification using infrared-differential interference contrast (IR-DIC) microscopy.

Whole cell patch-clamp mode was obtained by using patch pipettes with a resistance of 3-5 MΩ and an intracellular solution containing (in mM): K-gluconate 130, CaCl_2_ 1, EGTA 0.1, HEPES 10, MgATP 5, NaGTP 0.4, pH 7.25. Intracellular potentials were recorded in current clamp mode, amplified and low-pass filtered at 3 kHz using a multiclamp 700A amplifier (Axon/Molecular Devices, Biberach, Germany) and sampled at 20 kHz with a CED mikro1401 mkII ADC board and Spike2 software (CED, Cambridge, UK).

Data was analyzed using custom scripts in Spike2 and Matlab (MathWorks, Natick, MA, USA). Action potentials (AP) were only analyzed from cells showing APs with a peak above 0 mV. Action potentials without overshoot (> 0 mV) were excluded from analysis.

**Electrophysiology of primary *Ugcg*^f/f//NesCre^ neurons**

Whole cell recordings were performed at room temperature with 3-4 MΩ borosilicate glass pipettes filled with (in mM): K-gluconate 140, KCl 3, NaCl 4, EGTA 0.2, HEPES 10, MgATP 4, NaGTP 0.3, adjusted to pH 7.4 with KOH. The bath solution contained (in mM): NaCl 140, KCl 3, CaCl_2_ 2, MgCl_2_ 2, glucose 15, HEPES 10, adjusted to pH 7.3 with NaOH. Potentials were not corrected for liquid junction potential. Current and voltage signals were recorded with an EPC-7 amplifier (HEKA). Data were low-pass filtered at 3 kHz, amplified, and sampled with 20 kHz (Micro1401mkII, CED, Cambridge, UK). Stimulus delivery and data acquisition were performed using Signal3 software (CED). Cells were voltage clamped at -70 mV and passive membrane properties were measured with a 5 mV rectangular pulse. During current clamp recording, the neurons were kept at ~-70 mV through injection of a DC current. Action potentials were elicited with current injections in the range of 50 – 100 pA.

**Supplementary References**

1. Arch JR, Hislop D, Wang SJ, Speakman JR (2006) Some mathematical and technical issues in the measurement and interpretation of open-circuit indirect calorimetry in small animals. Int J Obes (Lond) 30: 1322-1331.

2. Drozdz A (1975) Food habits and food assimilation in mammals. In: Grodzinski W, Klekowski RZ, Duncan A, editors. Methods for Ecological Bioenergetics. Oxford, UK: Blackwell. pp. 23-47.

3. Brito MN, Brito NA, Baro DJ, Song CK, Bartness TJ (2007) Differential activation of the sympathetic innervation of adipose tissues by melanocortin receptor stimulation. Endocrinology 148: 5339-5347.

4. Chomczynski P, Sacchi N (1987) Single-step method of RNA isolation by acid guanidinium thiocyanate-phenol-chloroform extraction. Anal Biochem 162: 156-159.

5. Zolotukhin S, Byrne BJ, Mason E, Zolotukhin I, Potter M, et al. (1999) Recombinant adeno-associated virus purification using novel methods improves infectious titer and yield. Gene Ther 6: 973-985.

6. Jennemann R, Sandhoff R, Langbein L, Kaden S, Rothermel U, et al. (2007) Integrity and barrier function of the epidermis critically depend on glucosylceramide synthesis. J Biol Chem 282: 3083-3094.

7. Keppler A, Gretz N, Schmidt R, Kloetzer HM, Groene HJ, et al. (2007) Plasma creatinine determination in mice and rats: an enzymatic method compares favorably with a high-performance liquid chromatography assay. Kidney Int 71: 74-78.

8. Jennemann R, Sandhoff R, Wang S, Kiss E, Gretz N, et al. (2005) Cell-specific deletion of glucosylceramide synthase in brain leads to severe neural defects after birth. Proc Natl Acad Sci U S A 102: 12459-12464.
